# Benefits and Harms of Low-Dose Rivaroxaban in Asian Patients With Atrial Fibrillation: A Systematic Review and Meta-analysis of Real-World Studies

**DOI:** 10.3389/fphar.2021.642907

**Published:** 2021-05-28

**Authors:** Jun Qian, Yi-Dan Yan, Sheng-Yan Yang, Chi Zhang, Wen-Yan Li, Zhi-Chun Gu

**Affiliations:** ^1^ Department of Pharmacy, Gongli Hospital of Pudong New Area, Naval Medical University, Shanghai, China; ^2^ Department of Pharmacy, Renji Hospital, School of Medicine, Shanghai Jiaotong University, Shanghai, China; ^3^ Department of Pharmacy, Second Affiliated Hospital of Naval Medical University, Shanghai, China; ^4^ Shanghai Anticoagulation Pharmacist Alliance, Shanghai Pharmaceutical Association, Shanghai, China; ^5^ Chinese Society of Cardiothoracic and Vascular Anesthesiology, Beijing, China

**Keywords:** atrial fibrillation, direct oral anticoagulants, rivaroxaban, Asian, low-dose

## Abstract

**Background:** Low-dose prescription of rivaroxaban was common among patients with atrial fibrillation (AF) in Asia. However, the benefits and harms of rivaroxaban at a low dosage in Asian patients with AF remains unclear. Accordingly, we aimed to collect and summarize all available evidence to fill this important knowledge gap.

**Methods:** In this systematic review and meta-analysis, we systematically searched databases of MEDLINE, EMBASE, and Cochrane Library for relevant studies from inception until February 23, 2021. Eligible retrospective nationwide or health insurance database studies or prospective registration studies that reported efficacy (stroke/systemic embolism), safety (major bleeding, intracranial hemorrhage, gastrointestinal bleeding), or other outcomes (myocardial infarction, death) of low-dose rivaroxaban in comparison with warfarin in AF patients were enrolled. Data extraction and study quality assessment were conducted by two authors independently. Low dosing of rivaroxaban (15/10 mg) was defined as the received dose lower than the recommended dose (20 mg) approved in most districts. Hazard ratio (HR) with 95% confidence intervals (95% CIs) was pooled using a random-effect model. Subgroup analyses were conducted according to different dose regimens. Sensitivity analyses were conducted by sequential elimination of each study from the pool. Since potential effect modifiers (patient demographics, differences of each study, and others) may lead to bias in primacy outcomes, we performed a meta-regression analysis to explore the influence of these factors on the primary efficacy and safety outcomes.

**Results:** Totally, 12 studies involving 292,815 Asian patients with AF were included. All studies were detected as low to moderate risk bias. Low-dose rivaroxaban treatment in Asian AF patients was associated with a reduced risk of stroke/systemic embolism (HR: 0.76, 95% CI: 0.70–0.84, *I* ^*2*^: 57.8%), major bleeding (HR: 0.72, 95% CI: 0.62–0.84, *I* ^*2*^: 81.5%), and all-cause death (HR: 0.65, 95% CI: 0.58–0.73, *I* ^*2*^: 81.7%) when compared with warfarin. Furthermore, consistent results were observed among different dose regimens (10/15/20 mg) in all the clinical outcomes (*P*_interaction_ > 0.05 for each outcome). Meta-regression analysis failed to detect any potential confounding to impact the primacy outcomes.

**Conclusion:** Insights from the present meta-analysis, we found that low-dose rivaroxaban, even at a dosage of 10 mg daily, was associated with a reduced risk of stroke/SE and bleeding than warfarin in Asian AF patients. However, owing to considerable heterogeneity among included studies, further prospective studies are required to confirm these findings.

## Introduction

Direct oral anticoagulants (DOACs; rivaroxaban, apixaban, edoxaban, and dabigatran) have been shown to be as effective as and probably safer than vitamin K antagonists (VKAs) for the prevention of stroke/systemic embolism (SE) in atrial fibrillation (AF) (Steffel et al., 2018). Currently, the uptake of DOACs in AF patients is progressively growing. It is notable that off-label low-dosing prescriptions of DOACs in AF population are common in the clinical practice worldwide (23.2%), particularly in Asian countries (38.3%) (Camm et al., 2020). For Asian patients with AF, rivaroxaban is commonly prescribed, and its dose selection is more complicated. The subgroup analysis of the Rocket-AF trial, a global study investigating the efficacy and safety profiles of a daily regimen of 20 mg rivaroxaban in AF patients, indicated that the results from the Asian population did not differ from the western population (Wong et al., 2014). Another similar trial (J-ROCKET AF trial), comparing outcomes of low-dose rivaroxaban (15/10 mg once daily) with warfarin (target international normalized ratio: 1.5–2.5) in Japanese patients with AF, showed that patients using rivaroxaban had a similar risk in stroke/SE and major bleeding vs. those receiving warfarin (Hori et al., 2012). Currently, low-dose rivaroxaban (15 or 10 mg once daily) has only been approved for stroke prevention in AF patients in Japan and Taiwan. However, the benefit and risk profiles of low-dose rivaroxaban among Asian patients with AF remain unclear in daily practice. To fill this unmet knowledge gap, we collected and summarized all available real-world studies to assesses the benefits and harms of low-dose rivaroxaban in Asian AF population.

## Methods

### Literature Search

The present systematic review and meta-analysis was conducted according to the priori established protocol (PROSPERO: CRD42018089939) (Gu et al., 2020), and reported in line with the Preferred Reporting Items for Reporting Systematic Reviews and Meta-analyses (PRISMA) (Page et al., 2021). As for searching strategy, the first draft in Medline database was provided by a senior librarian, revised by three authors (Z.C, J. Q. and Y. Y.), iterated to generate the final version, and translated in other electronic databases. Finally, databases of Medline, Embase, and Cochrane Library were searched from inception to February 23, 2021 for relevant studies, with the restriction of English language and the following searching strategy: “atrial fibrillation” AND “rivaroxaban” or “Xarelto” AND “real-world” or “real world” or “population” or “nationwide” or “nation” or “registry” or “cohort” or “Medicare” or “claim”. In addition, any potential studies listed in retrieved articles were also identified.

### Study Selection

The eligible criteria for studies were as follows: 1) population: Asian patients with AF; 2) intervention: low-dosing of rivaroxaban (15/10 mg), defined as the received dose lower than the recommended dose approved in most districts (20 mg), except Japan and Taiwan; 3) comparison: VKAs; 4) outcomes: primary efficacy outcome (stroke/SE), primary safety outcomes (major bleeding, intracranial hemorrhage (ICH), and gastrointestinal (GI) bleeding) as defined in each study (Supplementary Tables S1, S2), secondary outcomes (myocardial infarction (MI) and death); 5) study design: real-world studies integrating data from electronic health records, claims databases, or disease registries; 6) follow-up duration: at least 12 weeks.

Studies that published only in the form of conference abstract were excluded. Two authors (J. Q. and Y. Y.) independently screened all titles and abstracts, and further assessed potentially eligible full-text based on entry criteria. The disagreements were resolved by discussion with the corresponding investigator (Z.C.).

### Data Extraction and Quality Assessment

Data were extracted by two authors (J. Q. and Y. Y.) independently, with the following items: study characteristics (the first author and publication year, Country or region/data source/inclusion period, inclusion and exclusion criteria, patient number in different treatment, adjusted method, period of follow-up, reported outcomes and their definition); demographics and clinical characteristics (age, sex, dosage of rivaroxaban, comorbidities, CHA2DS2-VASc score, HAS-BLED score, etc.); and data of efficacy and safety outcomes (stroke/SE, major bleeding, ICH, GI, MI, and death). The above information was extracted from articles and their supplementary materials, and only accessible data were analyzed.

Since real-world studies have a higher risk for bias relative to RCTs, we considered important factors in real-world study design and methods used to mitigate bias when comparing outcomes between rivaroxaban and comparator. NEW-Castle Ottawa scale (NOS) was used for Study quality assessment, with three items: Selection (representativeness of the exposed cohort, selection of the non exposed cohort, ascertainment of exposure, demonstration that outcome of interest was not present at start of study); Comparability (comparability of cohorts on the basis of the design or analysis); Outcome (assessment of outcome, was follow-up long enough for outcomes to occur, adequacy of follow up of cohorts) (Islam et al., 2018). A study can be awarded a maximum of one score for each numbered item within the Selection and Outcome categories, except Comparability (a maximum of two scores). The summary risk of bias was determined as low (NOS scores ≥7), moderate (4≤ NOS scores ≤6), and high (NOS scores ≤3).

### Statistical Analyses

Dichotomous variables were calculated as hazard risks (HRs) with 95% confidence intervals (95% CIs). The random effects model was used to assess the overall estimated effects, in which the uniformly minimum variance unbiased estimator of the average effect size was obtained by weighting each effect-size estimate by its inverse variance (Gu et al., 2020). When a single analysis involved > 2 studies, meta-analysis was performed. Heterogeneity among studies was evaluated using *I* ^2^ test, with *I* ^2^ > 50% representing considerable heterogeneity. Subgroup analyses were carried out based on different dose regimens (20/15/10 mg daily), with the interaction analyses (*p* for interaction) for estimating the comparability among dosage. Sensitivity analysis was performed to assess the robustness of the results by eliminating each study from the pool sequentially. Given that studies conducted in Japan and Taiwan where the lower dose is approved for AF treatment in general may differ from those conducted in Korea where the low dose is approved in those with renal impairment, further sensitivity analysis was conducted by excluding studies conducted in Korea. Because patients’ baseline characteristics (age, sex, dosage of rivaroxaban, comorbidities, CHA2DS2-VASc score, HAS-BLED score, etc.) may lead to bias on primacy outcomes, we performed a meta-regression analysis to explore the influence of these factors on outcomes. When a single analysis involved > 10 studies, publication bias was evaluated by employing funnel plots (Gu et al., 2020). All data were analyzed by using STATA version13.0 (Statacorp, College Station, Texas, United States), with *p* < 0.05 indicating a statistically significant difference. As all the included researches were real-world studies, the evidence quality was limited. More studies, especially high-quality trials, are required in the future.

## Results

### Study Selection and Characteristics

**FIGURE 1:**
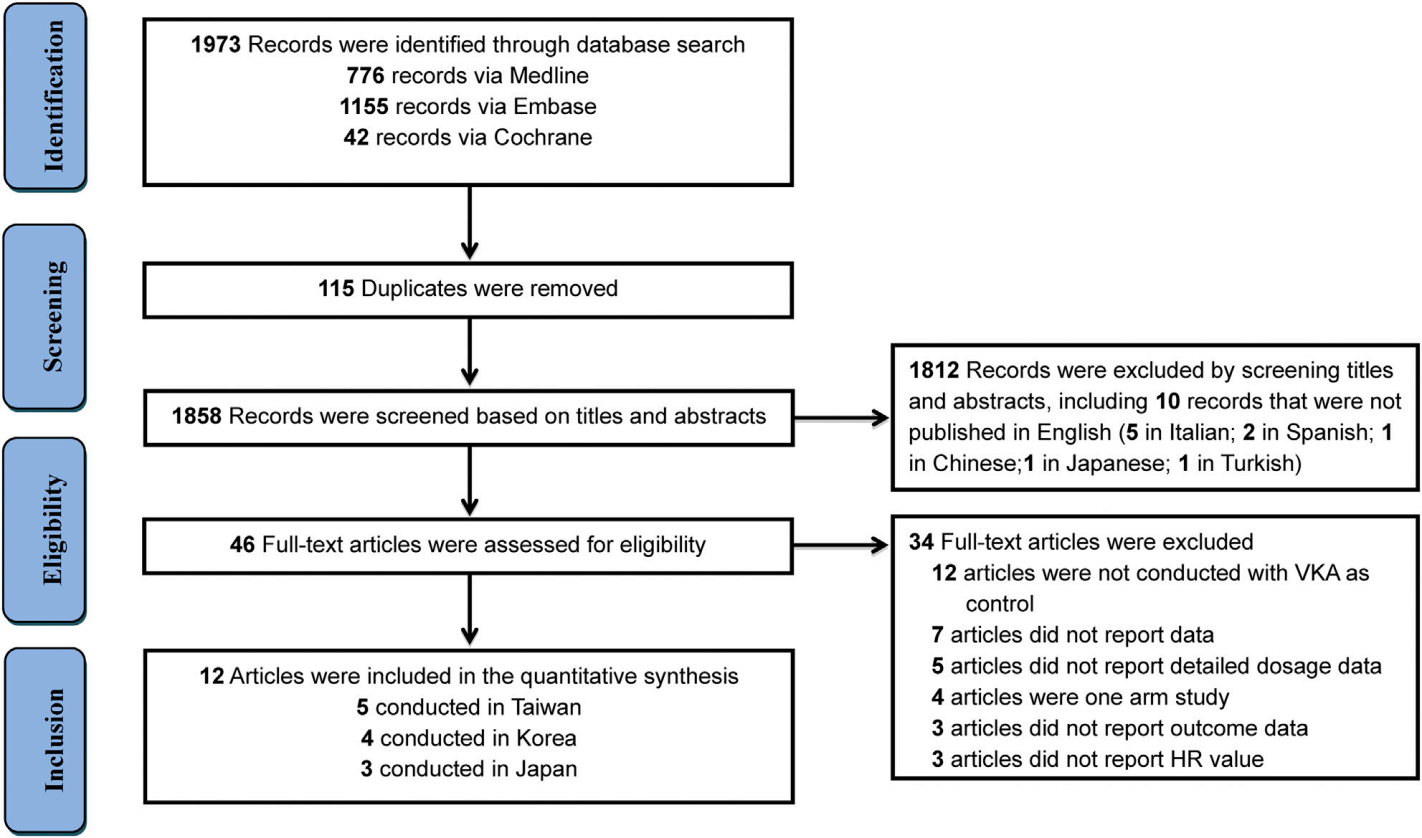
Flow diagram for the selection of eligible studies.

Totally, initial search yielded 1,973 records, among them 1,927 records were excluded due to duplication or irrelevance by screening titles and abstracts, including 10 records that were not published in English (5 in Italian; 2 in Spanish; 1 in Chinese; 1 in Japanese; 1 in Turkish) (Figure 1). 46 full-text articles were obtained for further assessment of eligibility, and 34 articles were excluded for reasons listed in Supplementary Table S3. Finally, 12 studies (Kohsaka et al., 2017; Chan et al., 2018; Cho et al., 2018; Huang et al., 2018; Lai et al., 2018; Lee et al., 2018; Okumura et al., 2018; Chan et al., 2019; Jeong et al., 2019; Lee et al., 2019; Cho et al., 2020; Kohsaka et al., 2020) involving 292,815 Asian patients with AF were included, with 169,383 patients using rivaroxaban and 123,432 patients using warfarin. Among them, eight studies with on-label low-dose rivaroxaban were conducted in Japan (3 studies) and Taiwan (5 studies), and four studies with off-label low-dose rivaroxaban were implemented in Korea (Table 1). Follow-up duration in these studies ranged from 11 to 24 months.

**TABLE 1:** Characteristics of included studies.

Study	Country or region/data source/inclusion period	Rivaroxaban numbers	Warfarin numbers	Adjusted method	Follow-up (months)	Reported outcomes
Kohsaka et al. (2017)	Japan/Health claims and Diagnosis Procedure Combination/2011.3.1-2016.3.31	10–15 mg/6,726	6,726	PSM	12	MB; Any bleeding
Okumura et al. (2018)	Japan/SAKURA AF Registry/2013.9.1-2015.12.31	10–15 mg/761	1,561	CA	24	Stroke/SE; ICH; MB; Death
Kohsaka et al. (2020)	Japan/Medical Data Vision Co Ltd database (372 acute care hospitals)/2011.3-2018.7	10–15 mg/17481	19,059	IPTW	NR	Stroke/SE; ICH; MB; GIB; Any bleeding
Cho et al. (2018)	Korea/National Health Insurance ServiceDatabase/2015.7.1-2016.12.31	10–15 mg/12399; 20 mg/8,601	10,409	PSA	15	Stroke/SE; MB; Death; Any bleeding
Lee et al. (2019)	Korea/National Health iInsurance Service database/2014.1-2016.12	15 mg/5,777; 20 mg/7,792	5,777; 7,792	PSM	17	Stroke; ICH; MB; GIB; Death
Jeong et al. (2019)	Korea/the department of Neurology and Cardiology at Chonnam National University Hospital in Gwangju/2014.1-2016.12	15 mg/414; 20 mg/390	804	PSM	At least 12	Stroke; ICH; MB; GIB; MI; Death; Any bleeding
Cho et al. (2020)	Korea/National Health Insurance service database/2015.7-2016.12	15 mg/4,879; 20 mg/4,760	4,536	PSA	15	Stroke/SE; MB; Death
Lee et al. (2018)	Taiwan/National Health Insurance Database/2013.2.1-2016.12.31	10 mg/11029; 15 mg/14971	16,000	PSA	15	Stroke/SE; ICH; MB; GIB; MI; Death
Chan et al. (2018)	Taiwan/National Health Insurance ResearchDatabase/2012.6.1-2016.12.31	10/15 mg 26,000; 20 mg/1777	19,375	PSA	15–18	Stroke/SE; ICH; MB; GIB; MI; Death
Huang et al. (2018)	Taiwan/National Health Insurance claims database/2012.6.1-2015.12.31	10 mg/3,104; 15 mg/5,996; 20 mg/1,509	9,637	PSM	11–14	Stroke; ICH; GIB
Lai et al. (2018)	Taiwan/National Health Insurance claims database/2011.1.1-2015.5.31	10–15 mg/1,207; 15 mg/788	1,207; 788	PSM	7	IS; ICH; GIB; MI; Death
Chan et al. (2019)	Taiwan/National Health Insurance Research Database/2012.6.1-2017.12.31	10/15 mg 31,108; 20 mg 1914	19,761	PSA	at least 16	IS/SE; ICH; MB; GIB; MI

Stroke/SE, stroke/systemic embolism; IS, ischemic stroke; MB, major bleeding; ICH, intracranial hemorrhage; GIB, gastrointestinal bleeding; MI, myocardial infarction; PSM, propensity score matching; CA, covariate adjustment; IPTW, inverse probability of treatment weighting; PSA, propensity score adjustment; NR, not reported.

### Patient Characteristics and Quality Assessment

Detailed patients and clinical characteristics are outlined in Supplementary Table S4. The mean age of patients was 74.2 years, and the proportion of male was 55.9%. The mean CHA2DS2-VASc score was 3.6, and mean HAS-BLED score was 2.7. The major comorbidities were hypertension (76.8%), followed by diabetes mellitus (35.5%), heart failure (21.9%), and previous stroke or TIA (21.3%).

Among included 12 trials, the summary risk of bias in nine studies (NOS scores: 7) was determined as low quality, and three studies was assessed as moderate quality (NOS scores: 6) (Supplementary Table S5).

### Efficacy and Safety of Low-Dose Rivaroxaban

Overall analyses on the efficacy and safety between low-dose rivaroxaban and warfarin in Asian patients with AF are presented in Table 2 and Supplementary Figures S1–S7. Considering the efficacy outcome, low-dose rivaroxaban was more effective than warfarin in reducing the risk of stroke/SE (11 studies, HR: 0.76, 95% CI: 0.70–0.84, *I* ^*2*^: 57.8%). When regarding primary safety, the use of low-dose rivaroxaban conferred a lower risk of major bleeding (10 studies, HR: 0.72, 95% CI: 0.62–0.84, *I* ^*2*^: 81.5%), ICH (8 studies, HR: 0.56, 95% CI: 0.46–0.68, *I* ^*2*^: 63.7%), as well as GIB (8 studies, HR: 0.81, 95% CI: 0.69–0.94, *I* ^*2*^: 58.5%) when compared with warfarin. Similarly, low-dose rivaroxaban was associated with a lower risk of MI (5 studies, HR: 0.73, 95% CI: 0.59–0.89, *I* ^*2*^: 7.0%) and all-cause death (8 studies, HR: 0.65, 95% CI: 0.58–0.73, *I* ^*2*^: 81.7%).

**TABLE 2:** Comparison of reduced-dose of Rivaroxaban vs. VKA.

Outcomes	Number of included studies	HR (95%CI)	*I* ^2^ (%)
Stroke/systemic embolism	11	0.76 (0.70–0.84)	57.8
Major bleeding	10	0.72 (0.62–0.84)	81.5
Intracranial hemorrhage	8	0.56 (0.46–0.68)	63.7
All-cause death	8	0.65 (0.58–0.73)	81.7
Gastrointestinal bleeding	8	0.81 (0.69–0.94)	58.5
Myocardial infarction	5	0.73 (0.59–0.89)	7.0
Any bleeding	4	0.96 (0.91–1.02)	28.7

HR, hazard ratio; CI, confidence interval.

### Subgroup Analyses Based on Dosage

**FIGURE 2:**
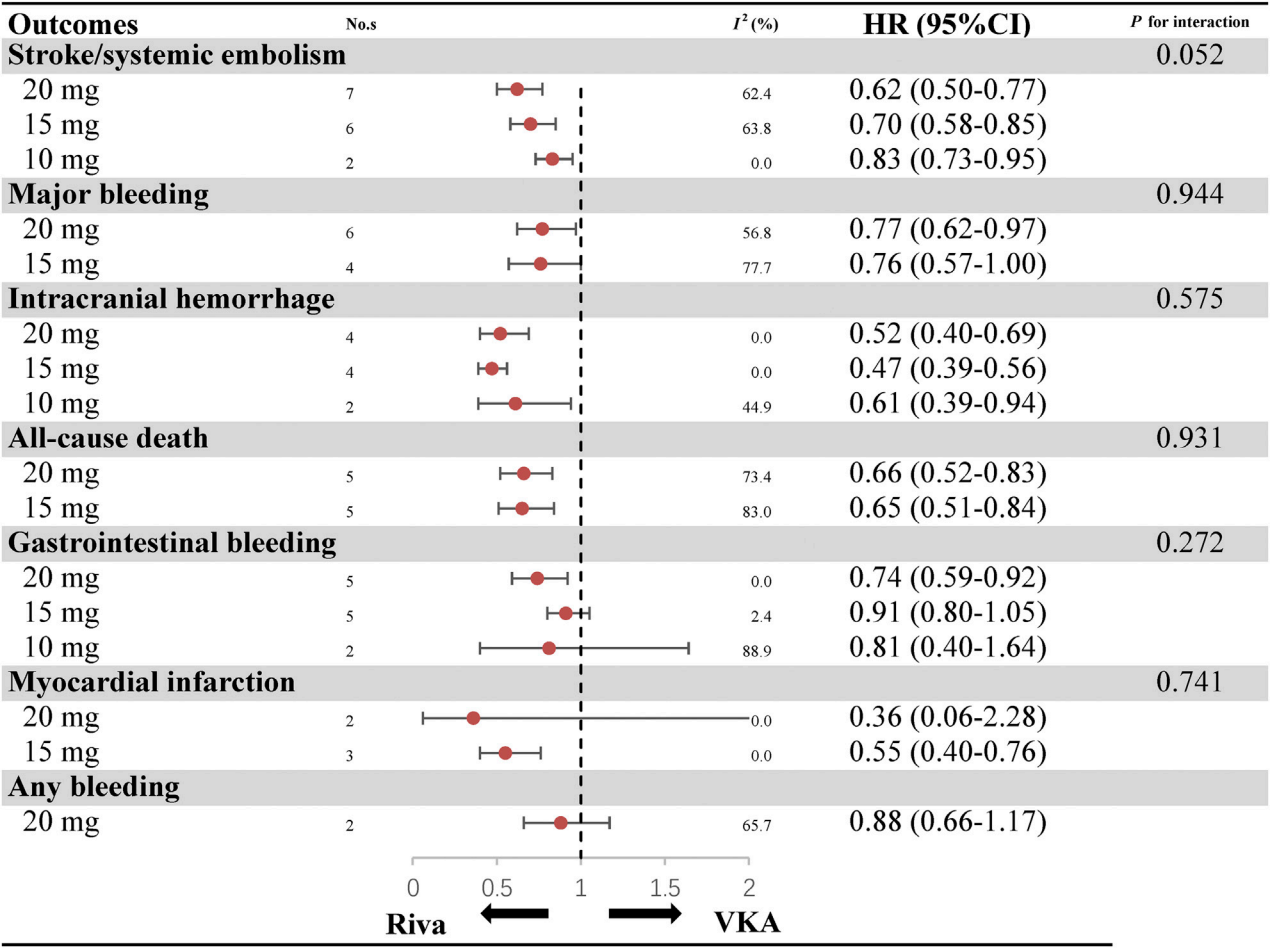
Benefits and harms of rivaroxaban by different dose regimen.

Analyses according to different dosage (20, 15, and 10 mg) are showed in Figure 2 and Supplementary Figures S8–S23. As for the efficacy, rivaroxaban resulted in a lower risk of stroke/SE than warfarin, regardless of 20 mg (7 studies, HR: 0.62, 95%CI: 0.50–0.77, *I* ^*2*^: 62.4%), 15 mg (6 studies, HR: 0.70, 95%CI: 0.58–0.85, *I* ^*2*^: 63.8%) and 10 mg (2 studies, HR: 0.83, 95%CI: 0.73–0.95, *I* ^*2*^: 0.0%). With respect to major bleeding risk, rivaroxaban 20 mg appeared reduced risk of major bleeding (6 studies, HR: 0.77, 95%CI: 0.62–0.97, *I* ^*2*^: 56.8%) and GIB (5 studies, HR: 0.74, 95%CI: 0.59–0.92, *I* ^*2*^: 0.0%) when compared to warfarin. However, this positive outcome was not observed in rivaroxaban 15 mg (4 studies, HR: 0.76, 95%CI: 0.57–1.00, *I* ^*2*^: 77.7% for MB; five studies, HR: 0.91, 95%CI: 0.80–1.05, *I* ^*2*^: 2.4% for GIB) and 10 mg (2 studies, HR: 0.81, 95%CI: 0.40–1.64, *I* ^*2*^: 88.9% for GIB). Besides, use of rivaroxaban 20 mg (4 studies, HR: 0.52, 95%CI: 0.40–0.69, *I* ^*2*^: 0.0% for ICH; five studies, HR: 0.66, 95%CI: 0.52–0.83, *I* ^*2*^: 73.4% for all-cause death), 15 mg (4 studies, HR: 0.47, 95%CI: 0.39–0.56, *I* ^*2*^: 0.0% for ICH; three studies, HR: 0.55, 95%CI: 0.40–0.76, *I* ^*2*^: 0.0% for MI; five studies, HR: 0.65, 95%CI: 0.51–0.84, *I* ^*2*^: 83.0% for all-cause death) and 10 mg (2 studies, HR: 0.61, 95%CI: 0.39–0.94, *I* ^*2*^: 44.9% for ICH) were consistently associated with decreased risk of ICH and all-cause death when compared to treatment with warfarin. The outcomes of major bleeding, myocardial infarction and all-cause death were not reported for rivaroxaban 10 mg. Interaction analyses revealed that results in effectiveness and safety were consistent among different dosage of rivaroxaban (*P*_interaction_ > 0.05 for each outcome).

### Sensitivity Analysis, Meta-regression Analysis, and Publication Bias

Sensitivity analyses were conducted by sequentially removing each study or by excluding studies conducted in Korea. The pooled results were in line with the primacy efficacy and safety outcomes (Supplementary Table S6). Also, meta-regression analysis failed to detect any potential confounding to impact the primacy outcomes (Supplementary Table S7). A visual inspection of funnel plots showed a relative symmetry, suggesting that the publication bias was not a concern overall (Supplementary Figure S24).

## Discussion

### Major Findings and Interpretation

The present study involved evidence from available real-world studies for evaluating the clinical outcomes of low-dose rivaroxaban among Asian patients with AF. Our results indicated that low-dose rivaroxaban was associated with a lowered risk of stroke/SE, bleeding (major bleeding, ICH, GIB), MI, and death compared with warfarin. In further analyses based on different dosages (20, 15, 10 mg), no significant difference was detected among groups in all the clinical outcomes. However, owing to relatively limited studies and considerable heterogeneity, further prospective studies are required to confirm these findings.

### Low-Dose Rivaroxaban Use in Asian Patients With AF

DOACs, including rivaroxaban, provide an alternative option to warfarin and are becoming the preferred anticoagulant choice for stroke prevention in most Asian AF patients. Reduced dose of rivaroxaban was approved by the National Food and Drug Administration for specific patients (those with low body weight, poor renal function or advanced age) (January et al., 2014; Steinberg et al., 2016). Given the association between higher bleeding risk and Asian population (Oh et al., 2016), physicians are more sensitive to the bleeding complications of anticoagulant therapy than to its effects in stroke prevention, generally trending to prescribe low-dose rivaroxaban for patients in clinical practice. Up to now, the data on effectiveness and safety of low-dose rivaroxaban in real-world remains uncertain. A nationwide Danish study suggested that low-dose use of DOACs might cause increased stroke risk (Nielsen et al., 2017). Another United States national prospective registry study indicated that low-dose DOACs were associated with an obvious increase in mortality (Steinberg et al., 2016). In recent years, many more contemporary real-world studies that focused on low-dose rivaroxaban in Asian have been published (Kohsaka et al., 2017; Chan et al., 2018; Cho et al., 2018; Huang et al., 2018; Lai et al., 2018; Lee et al., 2018; Okumura et al., 2018; Chan et al., 2019; Jeong et al., 2019; Lee et al., 2019; Cho et al., 2020; Kohsaka et al., 2020). It is well-known that real-world studies by integrating data from electronic health records (EHRs), claims databases, and disease registries could extend findings of RCTs to large patient populations in real-world practice. Accordingly, in the present study we summarized all available evidence from real-world studies for a comprehensive and rigorous meta-analysis on benefits and harms with low-dose rivaroxaban.

### The Efficacy and Safety of Low-Dose Rivaroxaban

Prior RCT trial (J-ROCKET) showed that low-dose rivaroxaban therapy was related to a marginal trend toward a reduction in stroke/SE (HR: 0.49, 95% CI: 0.24–1.00) and a similar risk in major bleeding (HR: 0.85, 95% CI: 0.50–1.43) vs. warfarin in Japanese patients (Hori et al., 2012). After pooling twelve real-world studies, our data showed that low-dose rivaroxaban was more effective and safer than warfarin for stroke prophylaxis in Asian patients. This difference might be partially explained by the control of time in therapeutic range (TTR), a critical indicator reflecting the anticoagulation effect for patients treated with warfarin and usually being well controlled during the management of RCTs. Nevertheless, TTR is hard to do well in real-world practice for multiple factors, such as frequent interactions with various diet or concomitant medications, genetic differences in metabolic or pharmacodynamic features among patients, insufficient management of anticoagulants, and lack of compliance in warfarin therapy, subsequently leading to poor clinical outcomes (Mitsuntisuk et al., 2020). Thus, Asian AF patients under warfarin treatment and with poor control of TTR might also be benefit from low-dose rivaroxaban.

### The Efficacy and Safety of Rivaroxaban at Different Dosages

Regarding different dosages, patients receiving rivaroxaban, even at a reduced dose of 10mg, carried a decreased risk of stroke/SE, ICH, death, and a comparative GIB risk compared to those taking warfarin, without discrepancy among subgroups. This was quite different from the results of increased stroke and death risks in Europeans and Americans treated with low-dose DOACs (Steinberg et al., 2016; Nielsen et al., 2017). Various outcomes probably due to patient-specific characteristics, especially the racial differences between Asians and non-Asians. According to the population pharmacokinetics-pharmacodynamics analyses, simulated steady-state exposure of Japanese AF patients treated with rivaroxaban 15 mg once daily (o.d.) approximates that achieved in simulated Caucasian AF patients treated with rivaroxaban 20 mg o. d. (Tanigawa et al., 2013). Besides, high prevalence of smaller body weight, advanced age, multiple underlying comorbidities and chronic kidney diseases in Asian AF patients also render them be benefited from low-dose rivaroxaban. Obviously, bodyweight is a vital factor affecting the risks for stroke/SE and bleeding when patients were under oral anticoagulant therapy. A retrospective cohort study suggested that being underweight was related to an increased risk of major bleeding and all-cause death compared with being normal weight or overweight in AF patients taking DOACs, whereas the risk of SE was similar (Park et al., 2017). For above evidence, it could be estimated that AF patients in Asia might need a lowered dose of rivaroxaban than those in western countries, and even rivaroxaban 10 mg remains a more effective and safe dose regimen than warfarin in the Asian population.

### Study Strengths and Limitations

Strength of this study is mainly that a systematic and rigorous approach was used to evaluate the benefits and risks of low-dose rivaroxaban in Asian patients with AF. We performed the subgroup analyses according to different dosages; applied interaction analyses to compare the difference among subgroups; conducted sensitivity analyses to strengthen the robustness of results. However, several limitations need to be acknowledged. First, included studies were conducted in different countries or regions, and the difference in the baseline characteristics of patients could not be excluded. To account for these issues, we have performed sensitivity analysis and meta-regression, without finding any potential confounding to impact the primacy outcomes. Moreover, the overall heterogeneity among the twelve studies was moderate, as revealed by *I* ^2^-values. Accordingly, a random-effect model was used in the statistical analysis. Second, real-world study has certain inevitable bias due to residual confounding and time-varying covariates/information censoring, which limited the generalization and extrapolation of results to clinical practice. Third, owing to the unavailability of detailed clinical information, we could not make a powerful subgroup analysis between patients who should be prescribed for standard-dose rivaroxaban and those who received under dosage for low body weight, poor renal function, or advanced age. Fourth, the TTR of patients in warfarin arm were not captured in this study, making the adequacy of the warfarin dosing schedules uncertain. This factor could have influenced the improved effectiveness of DOACs over warfarin that we observed, particularly in the low-dose group. Fifth, all included trials reported outcomes as defined in each study; however, it is not possible to evaluate whether small variations impact results obtained. Sixth, we did not search non-English articles and other electronic databases. However, we included studies identified in a comprehensive search of broad databases and are confident that this study covered the majority of studies. Lastly, due to the limited number of involved Asian districts, our results should be validated in further studies with AF patients from other Asian countries.

## Conclusion

In summary, low-dose rivaroxaban (15/10 mg), even the 10 mg-dose regimen, was associated with a reduced risk of stroke/SE and bleeding than warfarin among Asian patients with AF in the clinical setting. However, owing to considerable heterogeneity among included studies, further prospective studies are required to confirm these findings.

## Data Availability

The original contributions presented in the study are included in the article/Supplementary Material, further inquiries can be directed to the corresponding authors.

## References

[B1] Camm A. J. Cools F. Virdone S. Bassand J.-P. Fitzmaurice D. A. Arthur Fox K. A. (2020). Mortality in Patients with Atrial Fibrillation Receiving Nonrecommended Doses of Direct Oral Anticoagulants. J. Am. Coll. Cardiol. 76 (12), 1425–1436. 10.1016/j.jacc.2020.07.045 32943160

[B2] Chan Y.-H. Lee H.-F. See L.-C. Tu H.-T. Chao T.-F. Yeh Y.-H. (2019). Effectiveness and Safety of Four Direct Oral Anticoagulants in Asian Patients with Nonvalvular Atrial Fibrillation. Chest 156 (3), 529–543. 10.1016/j.chest.2019.04.108 31103697

[B3] Chan Y. H. See L. C. Tu H. T. Yeh Y. H. Chang S. H. Wu L. S. (2018). Efficacy and Safety of Apixaban, Dabigatran, Rivaroxaban, and Warfarin in Asians with Nonvalvular Atrial Fibrillation. Jaha 7 (8). 10.1161/JAHA.117.008150 PMC6015442 29622587

[B4] Cho M. S. Yun J. E. Park J. J. Kim Y. J. Lee J. Kim H. (2019). Outcomes after Use of Standard- and Low-Dose Non-vitamin K Oral Anticoagulants in Asian Patients with Atrial Fibrillation. Stroke 50, 110–118. 10.1161/strokeaha.118.023093 30580716

[B5] Cho M. S. Yun J. E. Park J. J. Kim Y. J. Lee J. Kim H. (2020). Pattern and Impact of Off-Label Underdosing of Non-vitamin K Antagonist Oral Anticoagulants in Patients with Atrial Fibrillation Who Are Indicated for Standard Dosing. Am. J. Cardiol. 125 (9), 1332–1338. 10.1016/j.amjcard.2020.01.044 32098658

[B6] Gu Z.-C. Wei A.-H. Zhang C. Wang X.-H. Zhang L. Shen L. (2020). Risk of Major Gastrointestinal Bleeding with New vs Conventional Oral Anticoagulants: A Systematic Review and Meta-Analysis. Clin. Gastroenterol. Hepatol. 18, 792–799. 10.1016/j.cgh.2019.05.056 31195162

[B7] Hori M. Matsumoto M. Tanahashi N. Momomura S.-i. Uchiyama S. Goto S. (2012). Rivaroxaban vs. Warfarin in Japanese Patients with Atrial Fibrillation. Circ. J. 76 (9), 2104–2111. 10.1253/circj.cj-12-0454 22664783

[B8] Huang H.-Y. Lin S.-Y. Cheng S.-H. Wang C.-C. (2018). Effectiveness and Safety of Different Rivaroxaban Dosage Regimens in Patients with Non-valvular Atrial Fibrillation: A Nationwide, Population-Based Cohort Study. Sci. Rep. 8 (1), 3451. 10.1038/s41598-018-21884-y 29472623 PMC5823875

[B9] Islam M. A. Khandker S. S. Alam F. Kamal M. A. Gan S. H. (2018). Genetic Risk Factors in Thrombotic Primary Antiphospholipid Syndrome: A Systematic Review with Bioinformatic Analyses. Autoimmun. Rev. 17 (3), 226–243. 10.1016/j.autrev.2017.10.014 29355608

[B10] January C. T. Wann L. S. Alpert J. S. Calkins H. Cigarroa J. E. Cleveland J. C. Jr. (2014). 2014 AHA/ACC/HRS Guideline for the Management of Patients with Atrial Fibrillation. J. Am. Coll. Cardiol. 64 (21), e1–e76. 10.1016/j.jacc.2014.03.022 24685669

[B11] Jeong H. K. Lee K. H. Park H. W. Yoon N. S. Kim M. C. Lee N. (2019). Real World Comparison of Rivaroxaban and Warfarin in Korean Patients with Atrial Fibrillation: Propensity Matching Cohort Analysis. Chonnam Med. J. 55 (1), 54–61. 10.4068/cmj.2019.55.1.54 30740341 PMC6351319

[B12] Kohsaka S. Katada J. Saito K. Jenkins A. Li B. Mardekian J. (2020). Safety and Effectiveness of Non-vitamin K Oral Anticoagulants versus Warfarin in Real-World Patients with Non-valvular Atrial Fibrillation: a Retrospective Analysis of Contemporary Japanese Administrative Claims Data. Open Heart 7 (1), e001232. 10.1136/openhrt-2019-001232 32341789 PMC7174060

[B13] Kohsaka S. Murata T. Izumi N. Katada J. Wang F. Terayama Y. (2017). Bleeding Risk of Apixaban, Dabigatran, and Low-Dose Rivaroxaban Compared with Warfarin in Japanese Patients with Non-valvular Atrial Fibrillation: a Propensity Matched Analysis of Administrative Claims Data. Curr. Med. Res. Opin. 33 (11), 1955–1963. 10.1080/03007995.2017.1374935 28857611

[B14] Lai C.-L. Chen H.-M. Liao M.-T. Lin T.-T. (2018). Dabigatran, Rivaroxaban, and Warfarin in the Oldest Adults with Atrial Fibrillation in Taiwan. J. Am. Geriatr. Soc. 66 (8), 1567–1574. 10.1111/jgs.15430 29975405

[B15] Lee H.-F. Chan Y.-H. Tu H.-T. Kuo C.-T. Yeh Y.-H. Chang S.-H. (2018). The Effectiveness and Safety of Low-Dose Rivaroxaban in Asians with Non-valvular Atrial Fibrillation. Int. J. Cardiol. 261, 78–83. 10.1016/j.ijcard.2018.03.063 29559181

[B16] Lee S.-R. Choi E.-K. Han K.-D. Jung J.-H. Oh S. Lip G. Y. H. (2019). Optimal Rivaroxaban Dose in Asian Patients with Atrial Fibrillation and Normal or Mildly Impaired Renal Function. Stroke 50 (5), 1140–1148. 10.1161/STROKEAHA.118.024210 30913984

[B17] Mitsuntisuk P. Nathisuwan S. Junpanichjaroen A. Wongcharoen W. Phrommintikul A. Wattanaruengchai P. (2020). Real‐World Comparative Effectiveness and Safety of Non‐Vitamin K Antagonist Oral Anticoagulants vs. Warfarin in a Developing Country. Clin. Pharmacol. Ther. 109, 1282–1292. 10.1002/cpt.2090 33113153

[B18] Nielsen P. B. Skjøth F. Søgaard M. Kjældgaard J. N. Lip G. Y. H. Larsen T. B. (2017). Effectiveness and Safety of Reduced Dose Non-vitamin K Antagonist Oral Anticoagulants and Warfarin in Patients with Atrial Fibrillation: Propensity Weighted Nationwide Cohort Study. Bmj 356, j510. 10.1136/bmj.j510 28188243 PMC5421446

[B19] Oh S. Goto S. Accetta G. Angchaisuksiri P. Camm A. J. Cools F. (2016). Vitamin K Antagonist Control in Patients with Atrial Fibrillation in Asia Compared with Other Regions of the World: Real-World Data from the GARFIELD-AF Registry. Int. J. Cardiol. 223, 543–547. 10.1016/j.ijcard.2016.08.236 27552578

[B20] Okumura Y. Yokoyama K. Matsumoto N. Tachibana E. Kuronuma K. Oiwa K. (2018). Three-Year Clinical Outcomes Associated with Warfarin vs. Direct Oral Anticoagulant Use Among Japanese Patients with Atrial Fibrillation ― Findings from the SAKURA AF Registry ―. Circ. J. 82 (10), 2500–2509. 10.1253/circj.CJ-18-0535 30078823

[B21] Page M. J. Moher D. Bossuyt P. M. Boutron I. Hoffmann T. C. Mulrow C. D. (2021). PRISMA 2020 Explanation and Elaboration: Updated Guidance and Exemplars for Reporting Systematic Reviews. Bmj 372, n160. 10.1136/bmj.n160 33781993 PMC8005925

[B22] Park C. S. Choi E.-K. Kim H. M. Lee S.-R. Cha M.-J. Oh S. (2017). Increased Risk of Major Bleeding in Underweight Patients with Atrial Fibrillation Who Were Prescribed Non-vitamin K Antagonist Oral Anticoagulants. Heart Rhythm 14 (4), 501–507. 10.1016/j.hrthm.2016.12.036 28042092

[B23] Steffel J. Verhamme P. Potpara T. S. Albaladejo P. Antz M. Desteghe L. (2018). The 2018 European Heart Rhythm Association Practical Guide on the Use of Non-vitamin K Antagonist Oral Anticoagulants in Patients with Atrial Fibrillation: Executive Summary. Europace 20 (8), 1231–1242. 10.1093/europace/euy054 29562331

[B24] Steinberg B. A. Shrader P. Thomas L. Ansell J. Fonarow G. C. Gersh B. J. (2016). Off-Label Dosing of Non-vitamin K Antagonist Oral Anticoagulants and Adverse Outcomes. J. Am. Coll. Cardiol. 68 (24), 2597–2604. 10.1016/j.jacc.2016.09.966 27978942

[B25] Tanigawa T. Kaneko M. Hashizume K. Kajikawa M. Ueda H. Tajiri M. (2013). Model-based Dose Selection for Phase III Rivaroxaban Study in Japanese Patients with Non-valvular Atrial Fibrillation. Drug Metab. Pharmacokinet. 28 (1), 59–70. 10.2133/dmpk.dmpk-12-rg-034 22813718

[B26] Wong K. S. L. Hu D. Y. Oomman A. Tan R.-S. Patel M. R. Singer D. E. (2014). Rivaroxaban for Stroke Prevention in East Asian Patients from the ROCKET AF Trial. Stroke 45 (6), 1739–1747. 10.1161/strokeaha.113.002968 24763930

