# Supplementary material for: Benefits and Harms of Low-Dose Rivaroxaban in Asian Patients With Atrial Fibrillation: A Systematic Review and Meta-analysis of Real-World Studies
Source: Front Pharmacol. 2021 May 28;12:642907. doi: 10.3389/fphar.2021.642907 (PMC8240755; doi:10.3389/fphar.2021.642907)
Supplement: Supplementary file 2 [file DataSheet1.docx]

SUPPLEMENTAL FILE

**Title:** Benefits and harms of low-dose rivaroxaban in Asians with atrial fibrillation: A systematic review and meta-analysis of real-world studies

**Contents**

**Indexs**

[Table S1. Outcome ascertainment and definition of outcome 3](#_Toc71535570)

[Table S2. Inclusion and exclusion criteria of the included studies 5](#_Toc71535571)

[Table S3. Excluded studies with reasons 9](#_Toc71535572)

[Table S4. Population Characteristics of the included studies 11](#_Toc71535573)

[Table S5. Quality assessment results of the included studies 12](#_Toc71535574)

[Table S6. Leave-1-out sensitivity analysis for reduced-dose of Rivaroxaban 13](#_Toc71535575)

[Table S7. Univariable meta-regression for reduced-dose of Rivaroxaban 14](#_Toc71535576)

[Figure S1. Stroke/systematic embolism with reduced-dose of Riva vs. VKA 16](#_Toc71535577)

[Figure S2. Major bleeding with reduced-dose of Riva vs. VKA 17](#_Toc71535578)

[Figure S3. Intracranial hemorrhage with reduced-dose of Riva vs. VKA 18](#_Toc71535579)

[Figure S4. All-cause mortality with reduced-dose of Riva vs. VKA 19](#_Toc71535580)

[Figure S5. GI bleeding with reduced-dose of Riva vs. VKA 20](#_Toc71535581)

[Figure S6. Myocardial infarction with reduced-dose of Riva vs. VKA 21](#_Toc71535582)

[Figure S7. Any bleeding with reduced-dose of Riva vs. VKA 22](#_Toc71535583)

[Figure S8. Stroke/systematic embolism with Riva 20mg vs. VKA 23](#_Toc71535584)

[Figure S9. Stroke/systematic embolism with Riva 15mg vs. VKA 24](#_Toc71535585)

[Figure S10. Stroke/systematic embolism with Riva 10mg vs. VKA 25](#_Toc71535586)

[Figure S11. Major bleeding with Riva 20mg vs. VKA 26](#_Toc71535587)

[Figure S12. Major bleeding with Riva 15mg vs. VKA 27](#_Toc71535588)

[Figure S13. Intracranial hemorrhage with Riva 20mg vs. VKA 28](#_Toc71535589)

[Figure S14. Intracranial hemorrhage with Riva 15mg vs. VKA 29](#_Toc71535590)

[Figure S15. Intracranial hemorrhage with Riva 10mg vs. VKA 30](#_Toc71535591)

[Figure S16. All-cause mortality with Riva 20mg vs. VKA 31](#_Toc71535592)

[Figure S17. All-cause mortality with Riva 15mg vs. VKA 32](#_Toc71535593)

[Figure S18. GI bleeding with Riva 20mg vs. VKA 33](#_Toc71535594)

[Figure S19. GI bleeding with Riva 15mg vs. VKA 34](#_Toc71535595)

[Figure S20. GI bleeding with Riva 10mg vs. VKA 35](#_Toc71535596)

[Figure S21. Myocardial infarction with Riva 20mg vs. VKA 36](#_Toc71535597)

[Figure S22. Myocardial infarction with Riva 15mg vs. VKA 37](#_Toc71535598)

[Figure S23. Any bleeding with Riva 20mg vs. VKA 38](#_Toc71535599)

[Figure S24. Publication bias for reduced-dose of Rivaroxaban (A. Stroke/SE; B. MB; C. ICH; D. GIB) 39](#_Toc71535600)

# Table S1. Outcome ascertainment and definition of outcome

| **Study** | **Outcome ascertainment/Definition** |
| --- | --- |
| Kohsaka 2017 | ICD-10/MB was defined as bleeding requiring hospitalisation; Any bleeding included all bleeding events identified by ICD-10 codes or disease codes. |
| Okumura 2018 | NR/MB was defined as a reduction in the hemoglobin concentration of at least 2 g/dL, transfusion of at least 2 units of blood, or symptomatic bleeding in a critical area or organ, and was specified as the safety endpoint. |
| Kohsaka 2020 | ICD-10/stroke and SE required hospitalisation; Stroke was defined as ischaemic or haemorrhagic stroke; MB was defined as bleeding requiring hospitalisation; Any bleeding event recorded after the index date regardless of severity or need for hospitalisation. |
| Cho 2018 | ICD-10/IS was diagnosed when the primary diagnostic code of hospitalization and concomitant imaging study (computed tomography or magnetic resonance imaging) was performed; SE was diagnosed when it was the principal diagnosis requiring hospitalization; MB was defined as fatal bleeding, bleeding necessitating hospitalization, or bleeding that occurred in critical sites (intracranial, intraspinal, intraocular, retroperitoneal, or intramuscular with compartment syndrome). |
| Lee 2019 | ICD-10/IS: Admission≥1 and brain imaging (CT or MRI) ≥1; ICH: Admission≥1 or RBC transfusion≥1; MB: Intracranial bleeding or gastrointestinal bleeding (ICH, admission≥1 or RBC transfusion≥1, GI bleeding, admission≥1 and RBC transfusion≥1); GIB: Admission≥1 and RBC transfusion≥1. |
| Jeong 2019 | NR/Stroke was defined as the sudden onset of focal neurologic deficit which was consistent with the territory of a major cerebral artery. It was classified as an ischemic, hemorrhagic, or transient ischemic attack; MB was defined according to the International Society on Thrombosis and Hemostasis criteria, as the decrease of hemoglobin levels by a 2 g/dL or the requirement of a transfusion of more than 2 units of packed red blood cells, occurring at a critical site, or leading to death. |
| Cho 2020 | ICD-10/IS was diagnosed using the primary diagnostic code for hospitalization with a concomitant imaging study (computed tomogram or magnetic resonance imaging); SE was defined when it was primary diagnosis of hospitalization; MB was defined when it was fatal, necessitating hospitalization, or occurred in critical sites (intracranial, intraspinal, intraocular, retroperitoneal, or intramuscular with compartment syndrome). |
| Lee 2018 | ICD-9 or ICD-10/ICH was defined with the use of codes for atraumatic hemorrhage; All MB events were defined as the total number of hospitalized events of ICH, major GIB, and other sites of critical bleeding; Major GIB was defined as a hospitalized primary code indicating bleeding in the gastrointestinal tract. |
| Chan 2018 | ICD-9 or ICD-10/Clinical diagnosis of IS was determined according to the compatible brain imaging; ICH was defined with the use of codes for atraumatic hemorrhage; All MB events were defined as the total hospitalized events of ICH, major GIB, and other critical site bleedings; Major GIB was defined as a hospitalized primary code indicating bleeding in the gastrointestinal tract. |
| Huang 2018 | ICD-9/NR |
| Lai 2018 | ICD-9 or ICD-10/NR |
| Chan 2019 | ICD-9 or ICD-10/NR |

SE: systemic embolism; IS: ischemic stroke; MB: major bleeding; ICH: intracranial hemorrhage; GIB: gastrointestinal bleeding; NR: not reported

# Table S2. Inclusion and exclusion criteria of the included studies

| **Study** | **Inclusion and exclusion criteria** |
| --- | --- |
| Kohsaka 2017 | Inclusion criteria were diagnosis of AF (ICD-10 code: I48) and prescription of one of the index OACs (apixaban, rivaroxaban, dabigatran or warfarin) after AF diagnosis. age 18 years or older and no use of any OAC during the baseline period (the 180 days before the index date).  Patients were excluded if they had a diagnosis of valvular AF (standard disease code: 8846941), post-operative AF (standard disease code: 884777), mechanical-valvular AF (ICD-10 code: T820) or rheumatic AF during the baseline period. Additional exclusion criteria were a diagnosis of hyperthyroidism or thyrotoxicosis (ICD-10 code: E05), procedures involving prosthetic heart valves performed, patients with end-stage renal disease, pregnancy, and other contraindicated patients. |
| Okumura 2018 | Inclusion criteria: (1) a diagnosis of non-valvular AF based on a 12-lead electrocardiography  (ECG) recording, 24-h Holter ECG recording, or event activated EGC recording; (2) age ≥20 years; and (3) treatment (either just initiated or already in place) with any anticoagulant drug for stroke prophylaxis. |
| Kohsaka 2020 | Inclusion criteria: diagnosis of AF at any time during the pre-index period and first prescription of any OAC (apixaban, dabigatran, edoxaban, rivaroxaban or warfarin) after a diagnosis of AF; age 18 years or older on the index date (defined as the date of the first prescription of any OAC); and no OAC prescription during the year preceding the index date (baseline period).  Exclusion criteria: valvular AF, postoperative AF, AF associated with mechanical valve malfunction, AF associated with mechanical complication of heart valve prosthesis or rheumatic AF, hyperthyroidism or thyrotoxicosis, those who underwent procedures involving prosthetic heart valves performed during the baseline period and those with haemodialysis or pregnancy. |
| Cho 2018 | Exclusion criteria: (1) prior use of any anticoagulants (including warfarin) before the start of the study period; (2) use of anticoagulants for <30 days; (3) use of ≥2 anticoagulants within 30 days; (4) CHA2DS2-VASc score <2; (5) mitral stenosis or prosthetic valve; (6) prior history of pulmonary thromboembolism or deep vein thrombosis; (7) prior joint replacement surgery; and (8) patients undergoing renal replacement therapy. |
| Lee 2019 | Exclusion criteria: patients ＜20 years old；diagnosed with valvular AF, PE, DVT, ESRD; with joint replacement, previous stroke, ICH or GIB |
| Jeong 2019 | The inclusion criteria were patients who were taking OACs whether warfarin or Rivaroxaban and having more than 2 points on the CHA2DS2-VASc score.  The exclusion criteria were valvular AF (rheumatic mitral stenosis, prosthetic mitral valve replacement or mitral valve repair), or any OAC class change (from warfarin to NOACs, from NOACs to warfarin). |
| Cho 2020 | Inclusion criteria: CHA2DS2-VASc score ≥2 points, nonvalvular AF, newly prescribed oral anticoagulants, either NOACs or warfarin  Exclusion criteria: (1) previous use of any anticoagulants before the study period, (2) irregular usage of anticoagulants (i.e., the use of anticoagulant for less than 30 days or switching of anticoagulants), (3) a CHA2DS2-VASc score <2, (4) the presence of mitral stenosis or prosthetic valve, (5) a prior history of pulmonary thromboembolism or deep vein thrombosis, (6) thromboprophylaxis after joint replacement surgery, (7) a lack of available data on weight or renal function, (8) the use of dabigatran, and (9) any dose-reduction criteria for NOAC use. |
| Lee 2018 | Exclusion criteria: We specifically focused on low-dose rivaroxaban in the present study. Patients were prescribed 20mg rivaroxaban once daily were excluded. Patients taking other NOACs (e.g. dabigatran, apixaban) anytime during the entire study period were also excluded. Additionally, since rivaroxaban was approved after February 1, 2013, those patients taking first dosage of warfarin before February 1, 2013 were also excluded, in order to achieve the head-to-head comparison with rivaroxaban. To establish a cohort of NVAF patients who took an oral anticoagulant for the primary purpose of ischemic stroke prevention, patients were excluded if they had diagnoses indicating venous thromboembolism (pulmonary embolism or deep vein thrombosis), valvular AF (mitral stenosis or history of valvular surgery), or required joint replacement therapy within 6months before the index date. Patients with end-stage renal disease were also excluded because NOACs are contraindicated in such patients in Taiwan. |
| Chan 2018 | Inclusion criteria:4 study groups (apixaban, dabigatran, rivaroxaban, and warfarin), AF, oral anticoagulants for the primary purpose of stroke prevention.  Exclusion Criteria：more than 1 kind of NOAC,  valvular AF (mitral stenosis or valvular surgery), venous thromboembolism (pulmonary embolism or deep vein thrombosis) or joint replacement, end-stage renal disease requiring renal replacement |
| Huang 2018 | Inclusion criteria: A retrospective cohort study with a new-user design. Patients who were at least 20 years old; had at least 1 inpatient or 2 separate outpatient diagnoses of AF, identified according to the ICD-9-CM code 427.31; and were prescribed rivaroxaban or warfarin from June 1, 2012 to December 31, 2015, fulfilled the inclusion criteria.  Exclusion criteria: had a prosthetic heart valve or mitral valve disease during the study period18, were pregnant, diagnosed with cancer, or under chronic dialysis within 12 months prior to the index date. |
| Lai 2018 | Inclusion criteria: adult aged 85 and older with a diagnosis of AF and flutter.  We also excluded individuals receiving two different kinds of study medications on the index date, those receiving concomitant antiplatelet therapy, those whose DOAC dosage could not be clarified, and those who had been exposed to warfarin between January 1, 2011, and May 31, 2012. |
| Chan 2019 | Exclusion criteria: Patients who took more than one DOAC during their treatment course were excluded from study. We also excluded patients diagnosed with end-stage renal disease, DVT, pulmonary embolism, joint replacement therapy, or valvular AF (eg, the diagnosis of mitral stenosis or valvular surgery) up to 6 months prior to the index date. |

# Table S3. Excluded studies with reasons

| **Study** | **Reason for exclusion** |
| --- | --- |
| Arihiro 2016(Arihiro et al., 2016) | Not reported data |
| Bando 2018(Bando et al., 2018) | Not reported low dose data |
| Cha 2017(Cha et al., 2017) | Not reported low dose data |
| Chan 2016(Chan et al., 2016) | Not reported low dose data |
| Chao 2020(Chao et al., 2020) | Not reported data |
| Cheng 2019(Cheng et al., 2019) | Not use warfarin as control |
| Cirrone 2018(Cirrone et al., 2018) | Not reported outcome data |
| Ikeda 2019(Ikeda et al., 2019a) | One arm study |
| Ikeda 2019(Ikeda et al., 2019b) | One arm study |
| Lai 2017(Lai et al., 2017) | Not use warfarin as control |
| Lee 2019(Lee et al., 2019a) | Not reported low dose data |
| Lee 2021(Lee et al., 2021) | Not reported data |
| Lee 2019(Lee et al., 2019b) | Not use warfarin as control |
| Lee 2019(Lee et al., 2019c) | Not use warfarin as control |
| Lee 2019(Lee and Lee, 2019) | Not reported outcome data |
| Li 2021(Li et al., 2021) | Not reported data |
| Li 2017(Li et al., 2017) | Not reported HR value |
| Lin 2018(Lin et al., 2018) | Not use warfarin as control |
| Meng 2019(Meng et al., 2019) | Not use warfarin as control |
| Mitsuntisuk 2020(Mitsuntisuk et al., 2020) | Not reported data |
| Murakawa 2020(Murakawa et al., 2020) | One arm study |
| Murata 2019(Murata et al., 2019) | Not reported data |
| Miyamoto 2020(Miyamoto et al., 2020) | One arm study |
| Saito 2020(Saito et al., 2020) | Not use warfarin as control |
| Shim 2020(Shim et al., 2020) | Not reported HR value |
| Shimokawa 2018(Shimokawa et al., 2018) | Not use warfarin as control |
| Sugrue 2020(Sugrue et al., 2020) | Not reported HR value |
| Tepper 2018(Tepper et al., 2018) | Not use warfarin as control |
| Tittl 2018(Tittl et al., 2018) | Not use warfarin as control |
| Tsai 2021(Tsai et al., 2021) | Not reported low dose data |
| Uchiyama 2019(Uchiyama et al., 2019) | Not use warfarin as control |
| Umei 2017(Umei et al., 2017) | Not use warfarin as control |
| Yamashita 2017(Yamashita et al., 2017) | Not reported data |
| Zhao 2020(Zhao et al., 2020) | Not reported outcome data |

# Table S4. Population Characteristics of the included studies

| **Study** | **Total number** | **Age** | **Male** | **HBP** | **DM** | **HF** | **MI** | **Renal disease** | **Liver disease** | **Bleeding history** | **Stroke or TIA** | **CHA2DS2-VASc score** | **HAS-BLED** | **Vascular disease** |
| --- | --- | --- | --- | --- | --- | --- | --- | --- | --- | --- | --- | --- | --- | --- |
| Kohsaka 2017 | 13452 | 75.8 | 62 | 53.8 | 28.9 | 35.3 | 2.9 | 4.4 | 10.6 | 11.2 | 22.3 | 3.3 | NR | 6.6 |
| Okumura 2018 | 2322 | 71.5 | 26.8 | 70 | 19.8 | 19.1 | NR | NR | NR | 1 | 10.3 | 2.87 | 1.32 | 12.6 |
| Kohsaka 2020 | 36540 | 76.2 | 61.1 | 54.9 | 30 | 37.1 | 3 | 7 | 12.5 | 12.2 | 21.2 | 3.8 | NR | 7.5 |
| Cho 2018 | 31409 | 72.8 | 51.9 | 87.4 | 46.5 | 21.3 | NR | 5 | NR | 1.1 | 23.3 | 3.6 | 2.5 | 11.9 |
| Lee 2019 | 27138 | 69.8 | 57.6 | 72.3 | 22.6 | 30.7 | 2.9 | NR | NR | NR | NR | 3.4 | NR | 17.5 |
| Jeong 2019 | 1608 | 71.1 | 60 | 58.2 | 22.9 | 9.3 | 8.6 | NR | NR | NR | 28.1 | 3.4 | NR | NR |
| Cho 2020 | 14175 | 68.7 | 60.3 | 88 | 47.7 | 19.7 | NR | 2 | NR | 0.9 | 20.8 | 2.4 | 1.8 | 11.4 |
| Lee 2018 | 42000 | 78 | 52 | 86 | 39 | 14 | 13 | 28 | 16 | 2 | 22 | 4.02 | 2.98 | NR |
| Chan 2018 | 47152 | 73.4 | 56.2 | 82.7 | 37.8 | 13.4 | 11.6 | 24 | 16 | 2 | 19.9 | 3.6 | 2.8 | NR |
| Huang 2018 | 20246 | 75.2 | 54.56 | 73.74 | 31 | 35.35 | 4.88 | 11.72 | 7.43 | 9.49 | 26.25 | 4.02 | 2.21 | NR |
| Lai 2018 | 3990 | 88.8 | 46.4 | 51.4 | 15.5 | 29 | 1.4 | NR | NR | 1.2 | 13.7 | 3.8 | NR | 4 |
| Chan 2019 | 52783 | 73.5 | 56.5 | 82 | 37.3 | 13.2 | NR | 25.5 | 15.6 | 2 | 19.9 | 3.6 | 2.8 | 0.1 |

HBP: high blood pressure; DM: diabetes mellitus; HF: heart failure; MI: myocardial infarction; TIA: transient ischemic attack; CHA2DS2-VASc=congestive heart failure, hypertension, age 75 years or older, diabetes mellitus, previous stroke/transient ischemic attack, vascular disease, age 65 to 74 years, female; HAS-BLED=hypertension, abnormal renal or liver function, stroke, bleeding history, labile INR, age 65 years or older, and antiplatelet drug or alcohol use. NR: not reported

# Table S5. Quality assessment results of the included studies

| **Study** | **Selection** | **Comparability** | **Outcome or exposure** | **NOS score** |
| --- | --- | --- | --- | --- |
| Kohsaka 2017 | 3 | 2 | 2 | 7 |
| Okumura 2018 | 3 | 2 | 2 | 7 |
| Kohsaka 2020 | 3 | 2 | 1 | 6 |
| Cho 2018 | 3 | 2 | 2 | 7 |
| Lee 2019 | 3 | 2 | 2 | 7 |
| Jeong 2019 | 2 | 2 | 2 | 6 |
| Cho 2020 | 3 | 2 | 2 | 7 |
| Lee 2018 | 3 | 2 | 2 | 7 |
| Chan 2018 | 3 | 2 | 2 | 7 |
| Huang 2018 | 3 | 2 | 2 | 7 |
| Lai 2018 | 3 | 2 | 1 | 6 |
| Chan 2019 | 3 | 2 | 2 | 7 |

NOS, NEW-Castle Ottawa scale; The summary risk of bias was determined as low (NOS scores≥7), moderate (4≤NOS scores≤6), and high (NOS scores≤3)

# Table S6. Leave-1-out sensitivity analysis for reduced-dose of Rivaroxaban

|  | **HR (95% CI), *I^2^*, N** | | | | | | |
| --- | --- | --- | --- | --- | --- | --- | --- |
| **Study omitted** | **Stroke/SE** | **MB** | **ICH** | **Death** | **GIB** | **MI** | **Any bleeding** |
| Kohsaka 2017 |  | 0.72 (0.62-0.85), 83.3%  (N=9) |  |  |  |  | 0.96 (0.89-1.04), 49.8%  (N=3) |
| Okumura 2018 | 0.75 (0.69-0.81), 38.7%  (N=10) | 0.70 (0.60-0.82), 81.4%  (N=9) | 0.50 (0.45-0.57), 0%  (N=8) | 0.64 (0.57-0.71), 77.3%  (N=7) |  |  |  |
| Kohsaka 2020 | 0.77 (0.70-0.86), 57.1%  (N=10) | 0.72 (0.61-0.85), 83.3%  (N=9) | 0.56 (0.45-0.70), 63.5%  (N=8) |  | 0.82 (0.69-0.98), 62.6%  (N=7) |  | 0.97 (0.88-1.06), 37.2%  (N=3) |
| Cho 2018 | 0.77 (0.70-0.86), 56.7%  (N=10) | 0.70 (0.60-0.80), 73.4%  (N=9) |  | 0.64 (0.57-0.72), 77.3%  (N=7) |  |  | 0.93 (0.88-0.99), 0%  (N=3) |
| Lee 2019 | 0.78 (0.71-0.86), 51.1%  (N=10) | 0.72 (0.61-0.85), 83.1%  (N=9) | 0.56 (0.45-0.69), 63.5%  (N=8) | 0.63 (0.57-0.70), 71.9%  (N=7) | 0.81 (0.68-0.97), 61.1%  (N=7) |  |  |
| Jeong 2019 | 0.77 (0.70-0.84), 58.3%  (N=10) | 0.73 (0.63-0.85), 82.9%  (N=9) | 0.56 (0.46-0.68), 59.7%  (N=8) | 0.66 (0.59-0.74), 82.0%  (N=7) | 0.83 (0.71-0.98), 64.0%  (N=7) | 0.72 (0.59-0.87), 3.2%  (N=4) | 0.97 (0.92-1.02), 19.3%  (N=3) |
| Cho 2020 | 0.79 (0.73-0.85), 41.3%  (N=10) | 0.70 (0.60-0.81), 80.2%  (N=9) |  | 0.66 (0.59-0.75), 81.9%  (N=7) |  |  |  |
| Lee 2018 | 0.75 (0.67-0.84), 55.3%  (N=10) | 0.76 (0.65-0.90), 75.5%  (N=9) | 0.59 (0.46-0.76), 60.0%  (N=8) | 0.70 (0.61-0.79), 67.7%  (N=7) | 0.89 (0.75-1.05), 41.1%  (N=7) | 0.76 (0.54-1.07), 0%  (N=4) |  |
| Chan 2018 | 0.76 (0.69-0.84), 58.3%  (N=10) | 0.74 (0.63-0.87), 81.8%  (N=9) | 0.57 (0.46-0.70), 63.2%  (N=8) | 0.67 (0.59-0.76), 81.4%  (N=7) | 0.86 (0.73-1.01), 58.5%  (N=7) | 0.72 (0.58-0.91), 8.4%  (N=4) |  |
| Huang 2018 | 0.77 (0.70-0.86), 61.0%  (N=10) |  | 0.56 (0.46-0.69), 61.4%  (N=8) |  | 0.78 (0.66-0.92), 56.0%  (N=7) |  |  |
| Lai 2018 | 0.76 (0.69-0.83), 59.4%  (N=10) |  | 0.56 (0.46-0.69), 67.6%  (N=8) | 0.66 (0.58-0.75), 84.0%  (N=7) | 0.81 (0.68-0.95), 63.1%  (N=7) | 0.73 (0.57-0.93), 25.2%  (N=4) |  |
| Chan 2019 | 0.77 (0.70-0.85), 58.3%  (N=10) | 0.74 (0.63-0.87), 82.1%  (N=9) | 0.56 (0.46-0.69), 63.7%  (N=8) |  | 0.85 (0.71-1.00), 62.5%  (N=7) | 0.71 (0.58-0.87), 1.4%  (N=4) |  |
| Excluding studies  conducted in Korea | 0.82 (0.76-0.89), 26.7%  (N=7) | 0.66 (0.57-0.75), 68.4%  (N=6) | 0.56 (0.45-0.69), 63.5%  (N=7) | 0.62 (0.55-0.68), 69.4%  (N=4) | 0.82 (0.68-0.98), 65.3%  (N=6) | 0.72 (0.59-0.87), 3.2%  (N=4) | 0.94 (0.89-0.99), 0%  (N=2) |

CI: confidence interval; Stroke/SE: stroke/systemic embolism; MB: major bleeding; ICH: intracranial hemorrhage; GIB: gastrointestinal bleeding; MI: myocardial infarction; N: number of studies

# Table S7. Univariable meta-regression for reduced-dose of Rivaroxaban

|  | **regression coefficients (95% CI), *P* value** | | | | | | |
| --- | --- | --- | --- | --- | --- | --- | --- |
| **Variable** | **Stroke/SE** | **MB** | **ICH** | **Death** | **GIB** | **MI** | **Any bleeding** |
| Age | 0.998 (0.98-1.02), 0.839 | 0.994 (0.97-1.02), 0.596 | 0.998 (0.96-1.04), 0.904 | 0.998 (0.98-1.01), 0.714 | 0.999 (0.97-1.03), 0.949 | 0.999 (0.91-1.09), 0.975 | 0.998 (0.94-1.06), 0.889 |
| Male | 1.000 (0.99-1.01), 0.958 | 1.001 (0.99-1.01), 0.89 | 1.000 (0.99-1.01), 0.992 | 0.999 (0.99-1.01), 0.848 | 1.003 (0.97-1.04), 0.827 | 1.013 (0.90-1.14), 0.768 | 1.001 (0.98-1.02), 0.879 |
| HBP | 0.998 (0.99-1.00), 0.554 | 0.999 (0.99-1.00), 0.707 | 0.998 (0.99-1.01), 0.657 | 0.997 (0.99-1.00), 0.388 | 0.997 (0.988-1.01)  0.588 | 0.986 (0.95-1.03), 0.386 | 1.000 (0.99-1.01), 0.886 |
| DM | 0.998 (0.99-1.01), 0.540 | 0.999 (0.99-1.01), 0.719 | 0.996 (0.98-1.01), 0.62 | 0.998 (0.99-1.00), 0.535 | 0.996 (0.98-1.01), 0.576 | 0.981 (0.92-1.04), 0.411 | 0.999 (0.99-1.01), 0.812 |
| HF | 1.001 (0.99-1.01), 0.695 | 1.000 (0.99-1.01), 0.924 | 1.003 (0.99-1.02), 0.641 | 1.003 (0.99-1.01), 0.532 | 1.001 (0.99-1.01), 0.827 | 1.014 (0.92-1.12), 0.707 | 0.999 (0.99-1.01), 0.857 |
| MI | 0.995 (0.98-1.01), 0.531 | 0.997 (0.98-1.02), 0.705 | 0.992 (0.96-1.02), 0.561 | 0.995 (0.97-1.01), 0.489 |  | 0.963 (0.83-1.11), 0.465 | 1.023 (0.69-1.52), 0.594 |
| Renal disease | 0.999 (0.99-1.01), 0.791 | 0.999 (0.99-1.01), 0.83 | 0.997 (0.98-1.02), 0.672 | 0.999 (0.99-1.01), 0.646 | 0.998 (0.98-1.01), 0.756 | 0.973 (0.74-1.28), 0.708 | 0.995 (0.76-1.29), 0.844 |
| Liver disease | 0.992 (0.96-1.03), 0.565 | 0.993 (0.93-1.06), 0.779 | 0.983 (0.92-1.05), 0.526 |  | 0.995 (0.96-1.04), 0.768 | 0.72 (0.02-24.58), 0.727 |  |
| Bleeding history | 1.002 (0.98-1.02), 0.799 | 1.001 (0.98-1.02), 0.903 | 1.004 (0.97-1.04), 0.795 | 0.955 (0.82-1.11), 0.469 | 1.002 (0.97-1.03), 0.867 | 0.66 (0.08-5.53), 0.578 | 1.000 (0.95-1.06), 0.956 |
| Stroke or TIA | 0.999 (0.98-1.02), 0.886 | 0.998 (0.98-1.02), 0.859 | 0.998 (0.96-1.03), 0.920 | 0.996 (0.98-1.01), 0.591 | 0.998 (0.95-1.04), 0.936 | 0.979 (0.85-1.13), 0.713 | 1.010 (0.93-1.09), 0.653 |
| CHA_2_DS_2_-VASc score | 0.971 (0.84-1.13), 0.669 | 0.953 (0.81-1.13), 0.536 | 0.961 (0.67-1.37), 0.804 | 0.94 (0.81-1.09), 0.36 | 0.907 (0.56-1.47), 0.653 | 0.681 (0.16-2.83), 0.496 | 0.936 (0.53-1.65), 0.665 |
| HAS-BLED | 0.967 (0.83-1.12), 0.609 | 0.974 (0.82-1.16), 0.718 | 0.954 (0.71-1.28), 0.694 | 0.958 (0.82-1.12), 0.486 | 0.932 (0.57-1.53), 0.713 | 0.544 (0-122.68), 0.677 |  |
| Vascular disease | 0.997 (0.97-1.02), 0.771 | 0.998 (0.97-1.02), 0.819 | 0.996 (0.95-1.05), 0.793 | 0.997 (0.96-1.03), 0.798 |  |  | 0.999 (0.88-1.13), 0.909 |

CI: confidence interval; Stroke/SE: stroke/systemic embolism; MB: major bleeding; ICH: intracranial hemorrhage; GIB: gastrointestinal bleeding; MI: myocardial infarction; TIA: transient ischemic attack


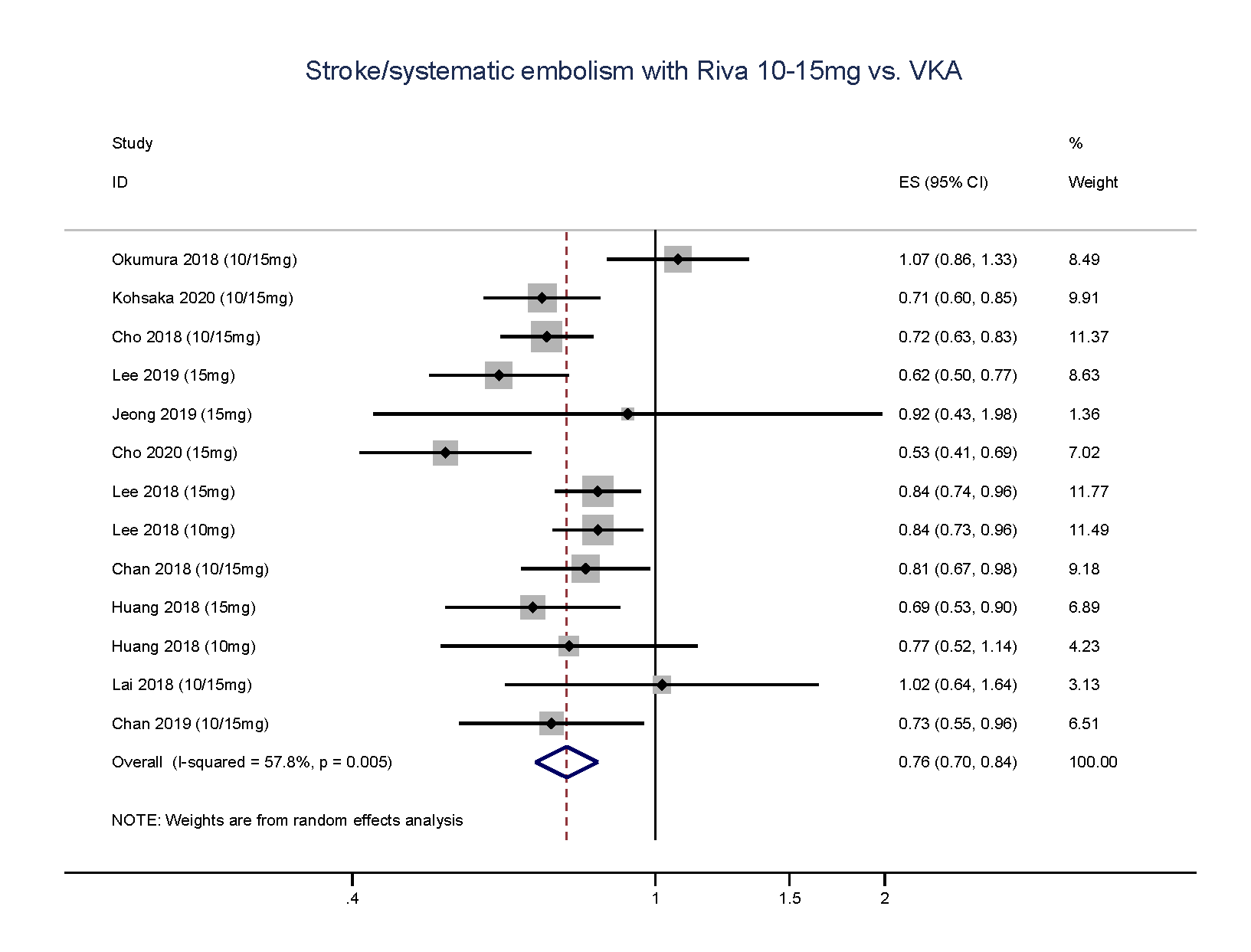


# Figure S1. Stroke/systematic embolism with reduced-dose of Riva vs. VKA (ES indicates hazard ratio)


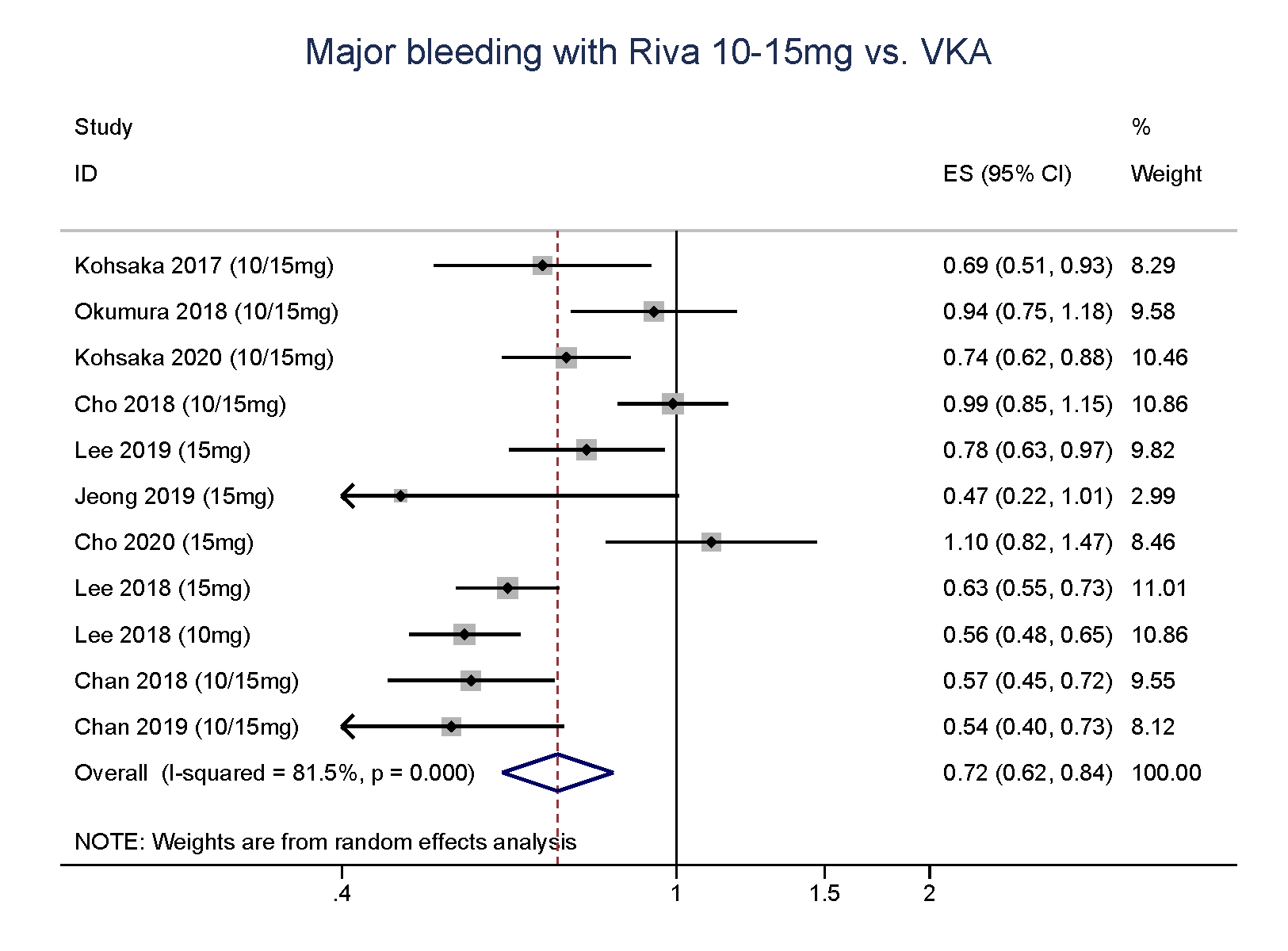


# Figure S2. Major bleeding with reduced-dose of Riva vs. VKA (ES indicates hazard ratio)


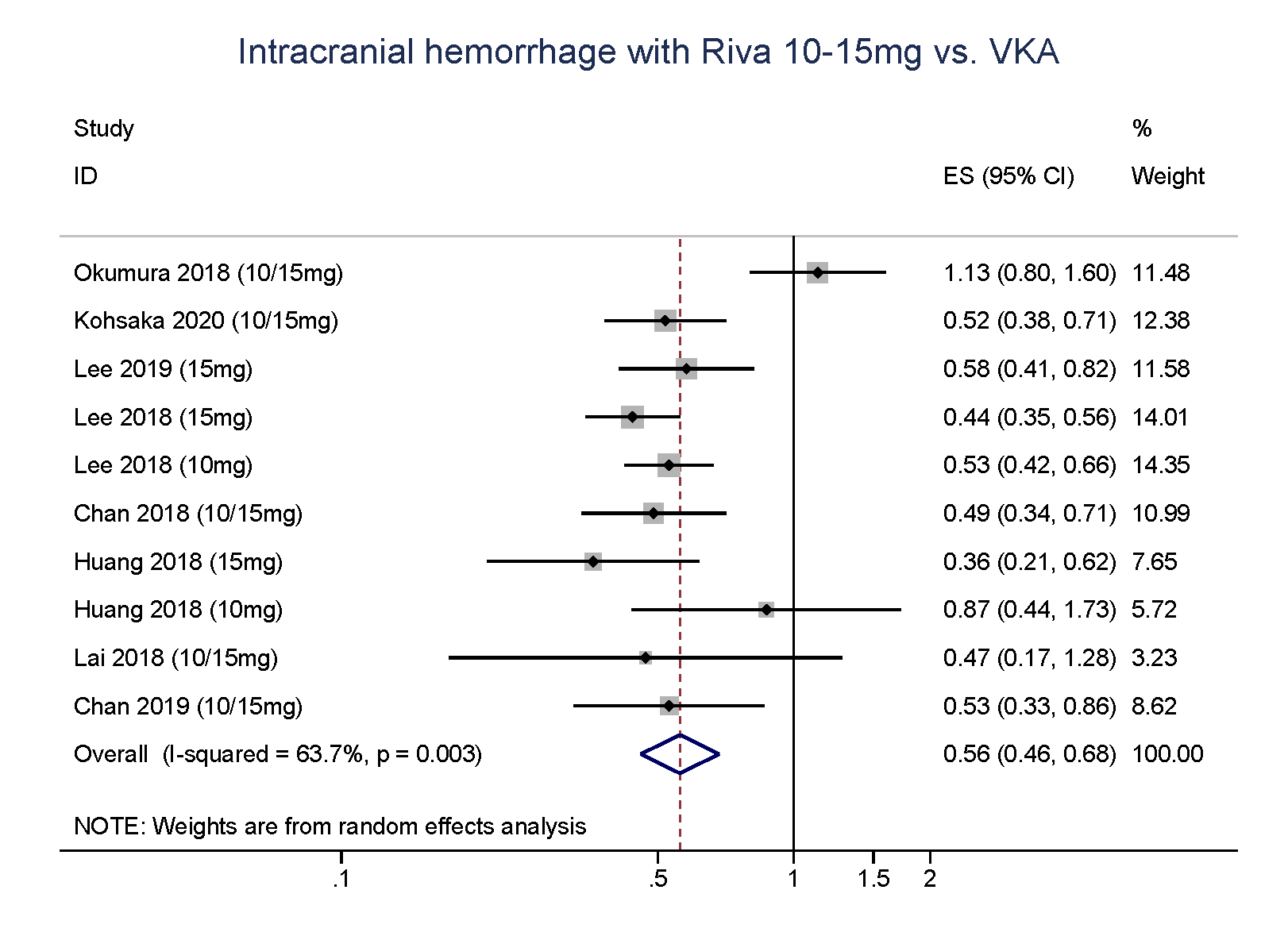


# Figure S3. Intracranial hemorrhage with reduced-dose of Riva vs. VKA (ES indicates hazard ratio)


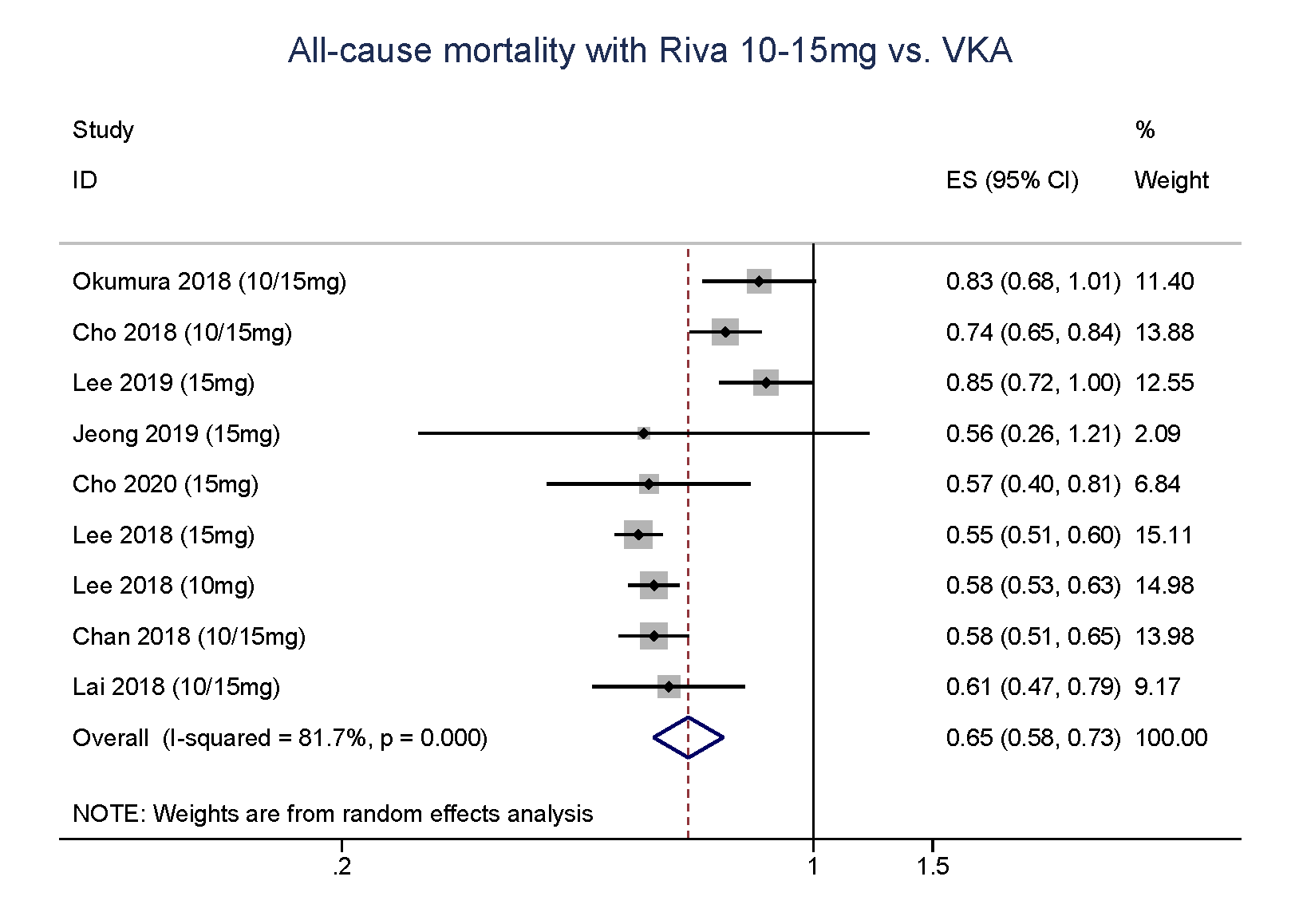


# Figure S4. All-cause mortality with reduced-dose of Riva vs. VKA (ES indicates hazard ratio)


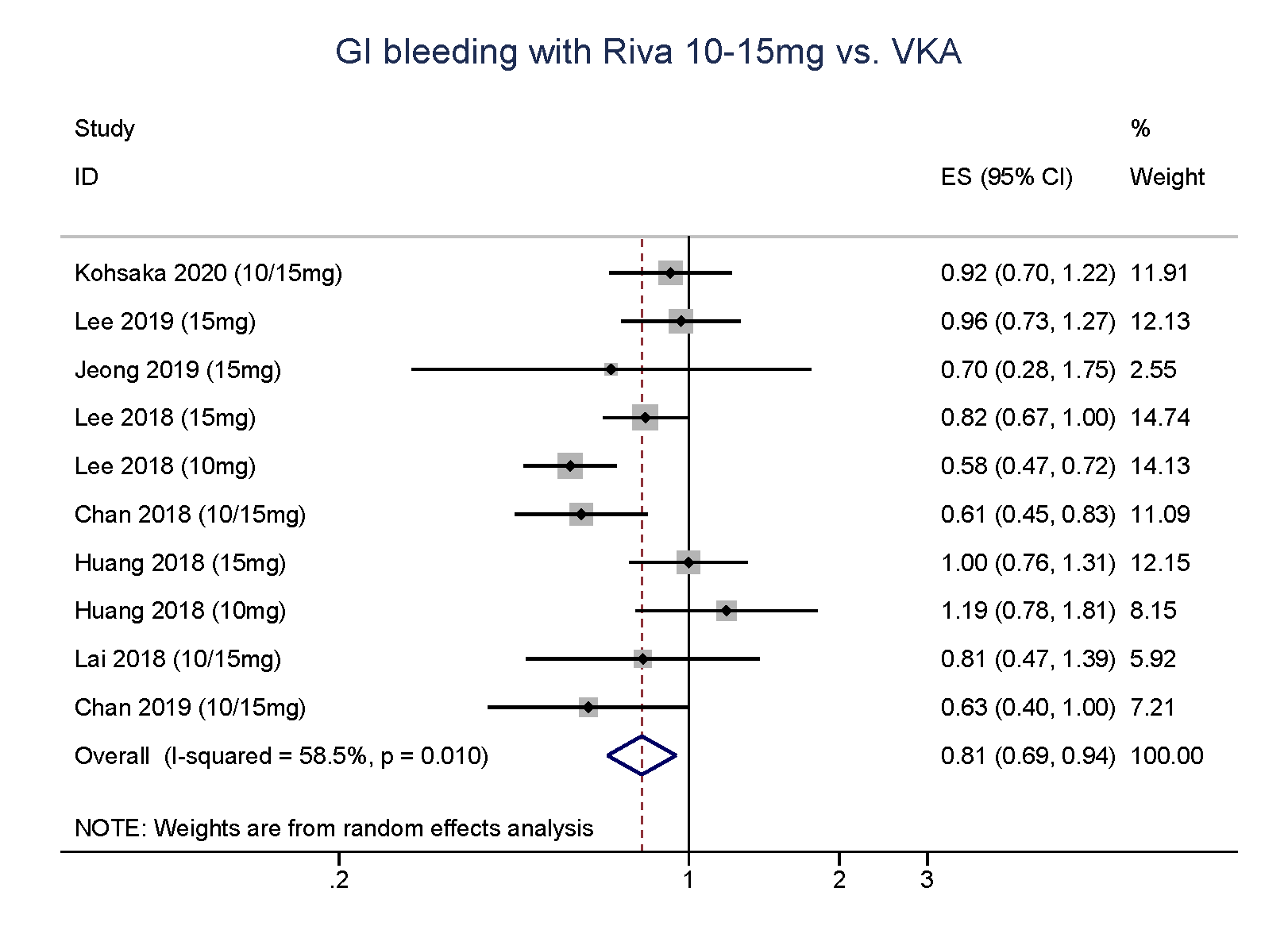


# Figure S5. GI bleeding with reduced-dose of Riva vs. VKA (ES indicates hazard ratio)


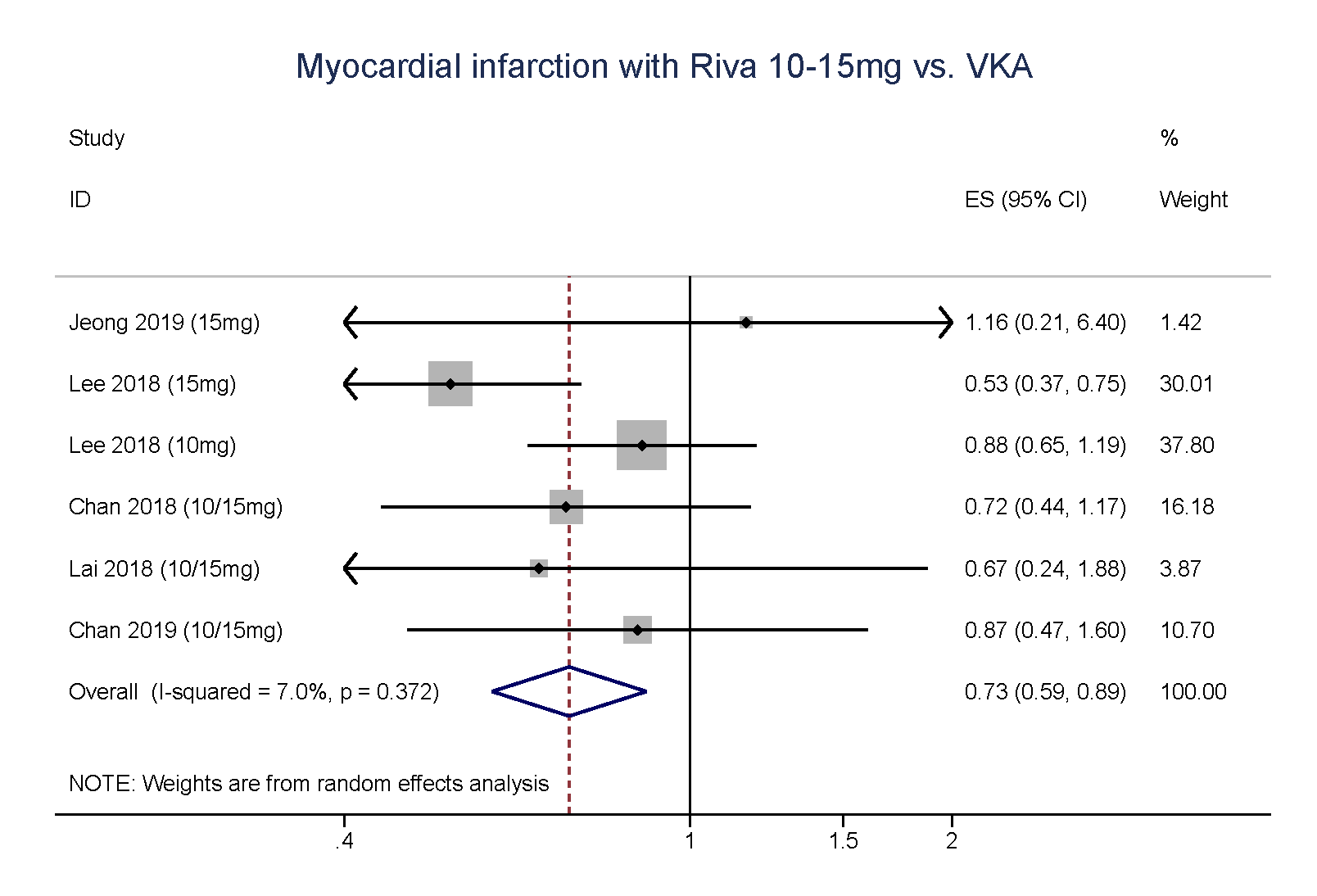


# Figure S6. Myocardial infarction with reduced-dose of Riva vs. VKA (ES indicates hazard ratio)


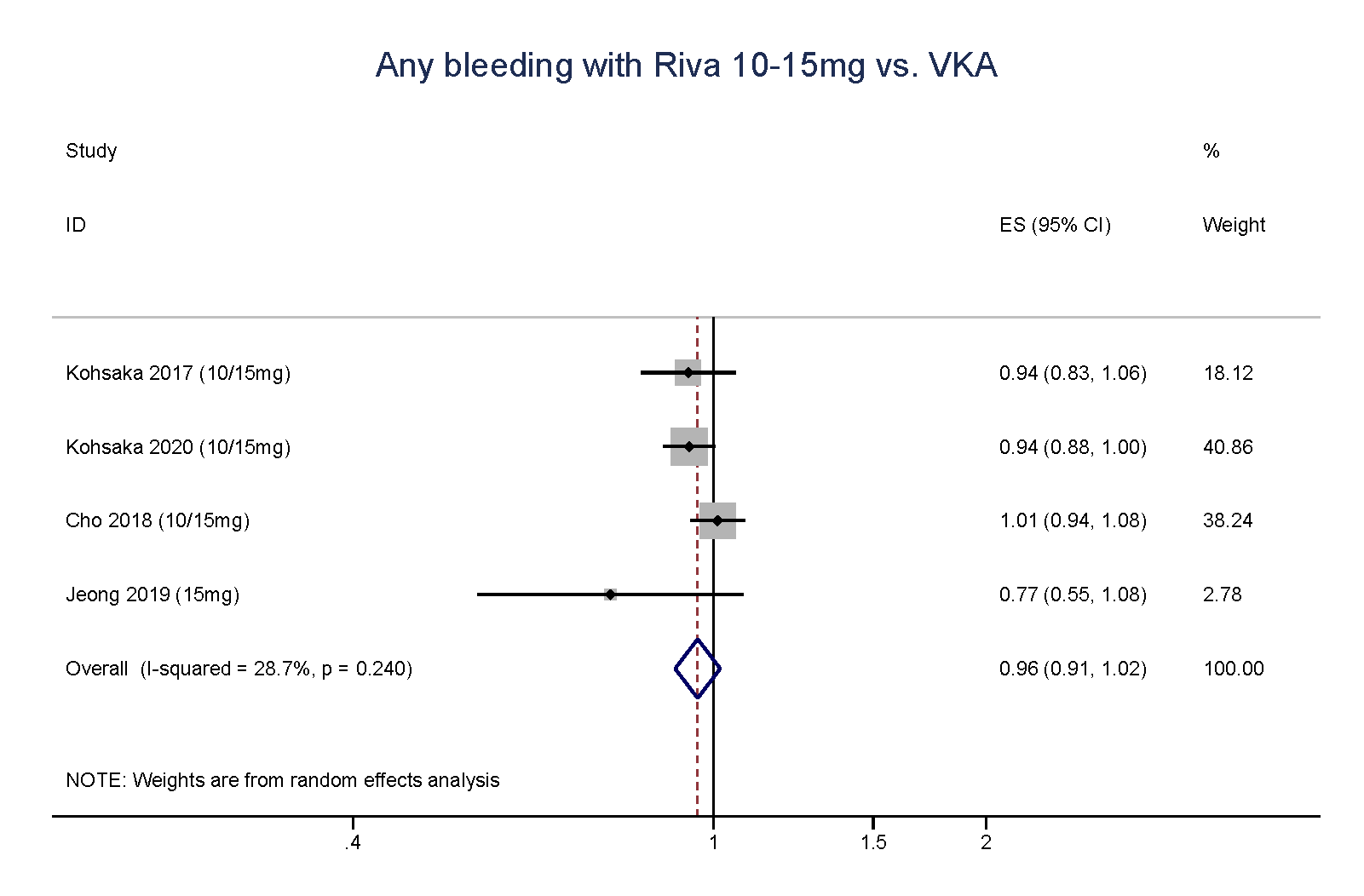


# Figure S7. Any bleeding with reduced-dose of Riva vs. VKA (ES indicates hazard ratio)


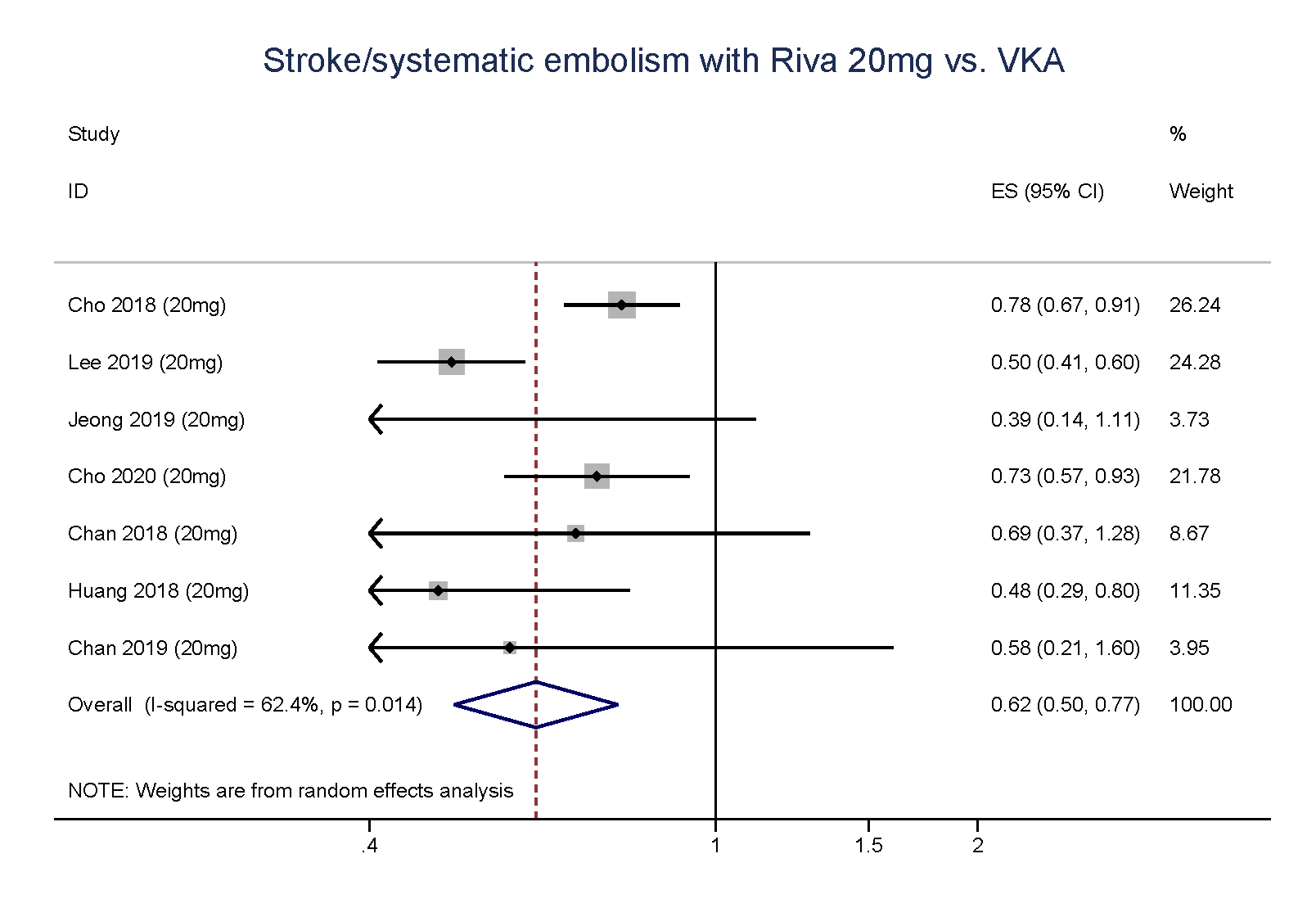


# Figure S8. Stroke/systematic embolism with Riva 20mg vs. VKA (ES indicates hazard ratio)


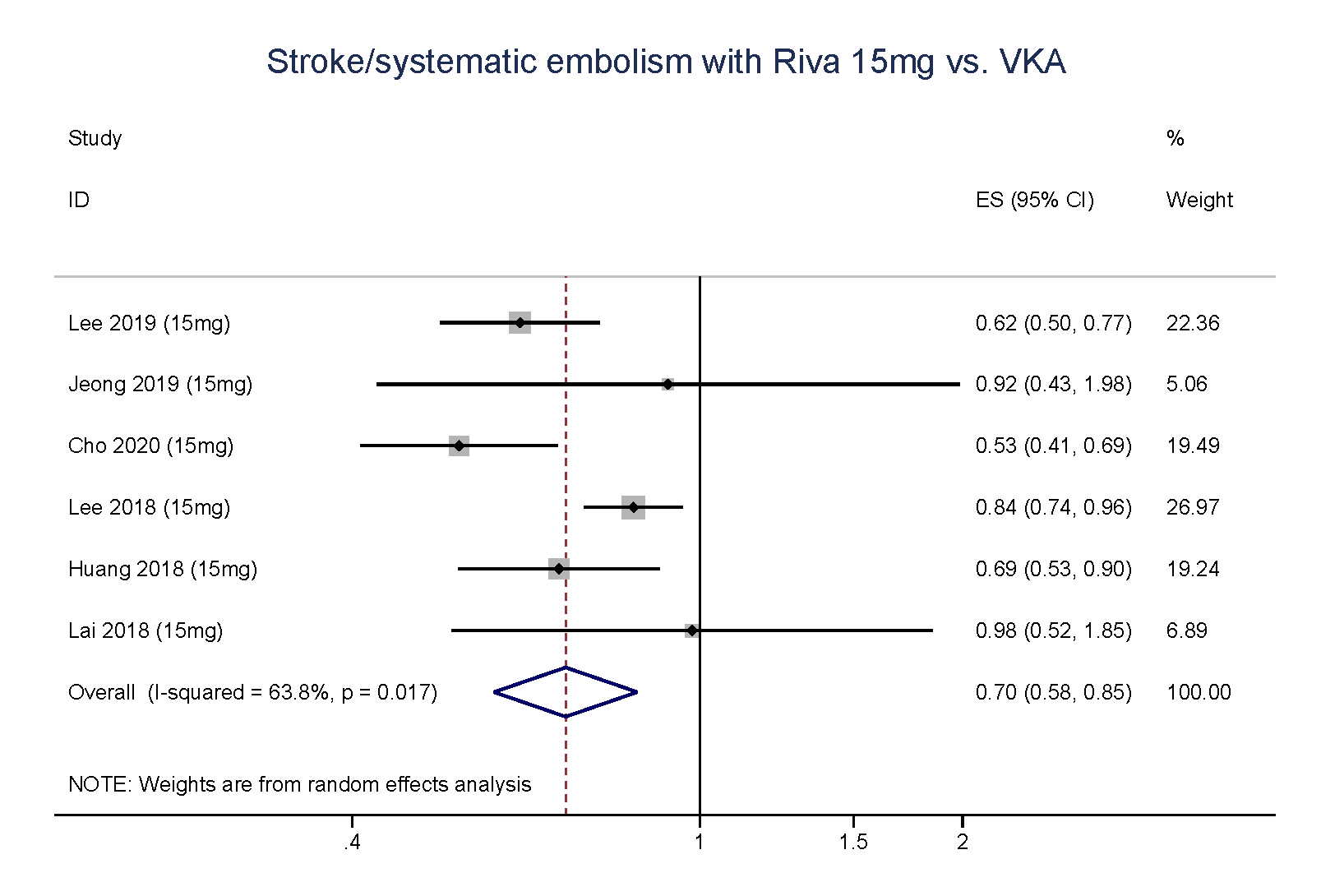


# Figure S9. Stroke/systematic embolism with Riva 15mg vs. VKA (ES indicates hazard ratio)


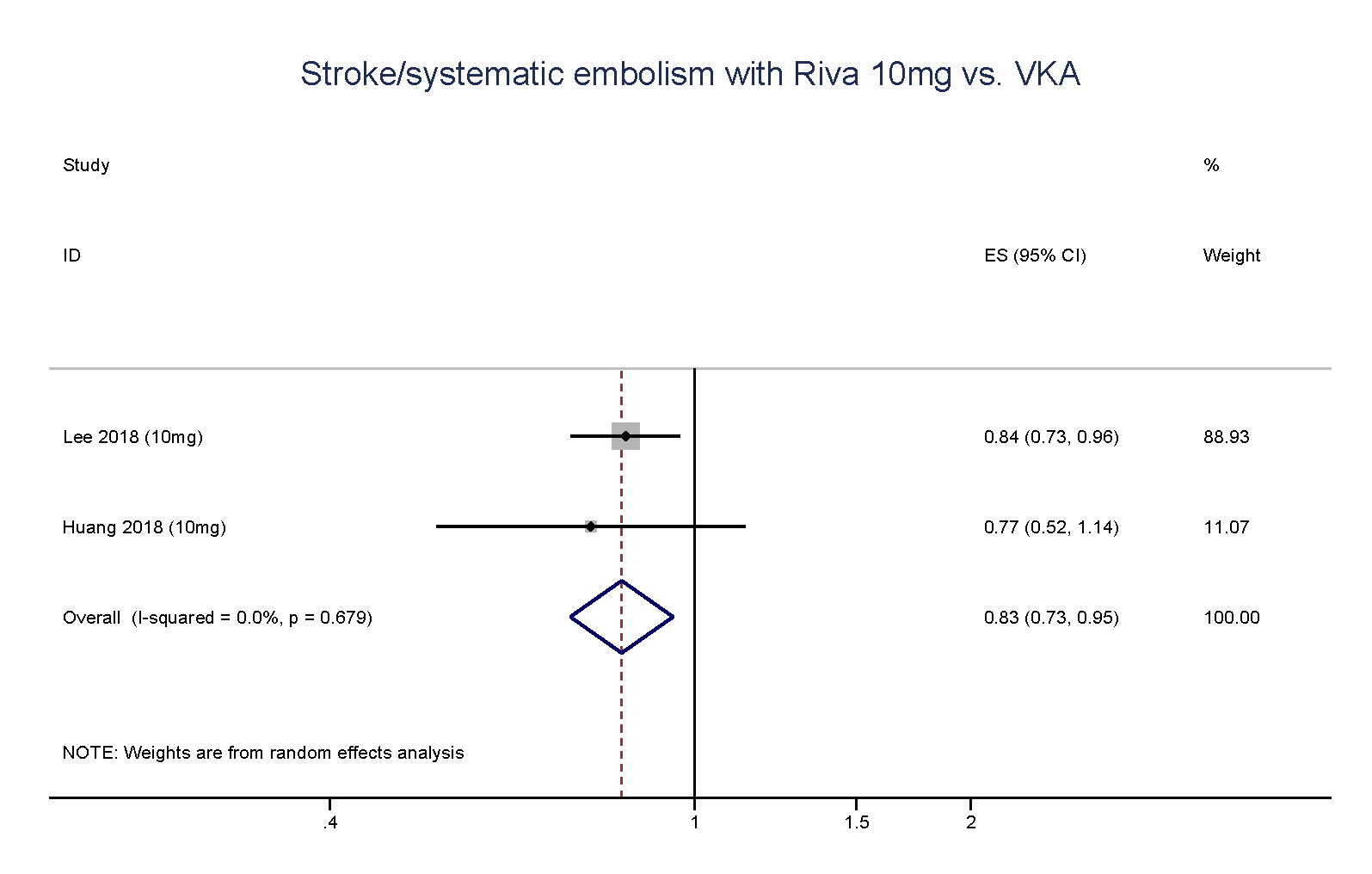


# Figure S10. Stroke/systematic embolism with Riva 10mg vs. VKA (ES indicates hazard ratio)


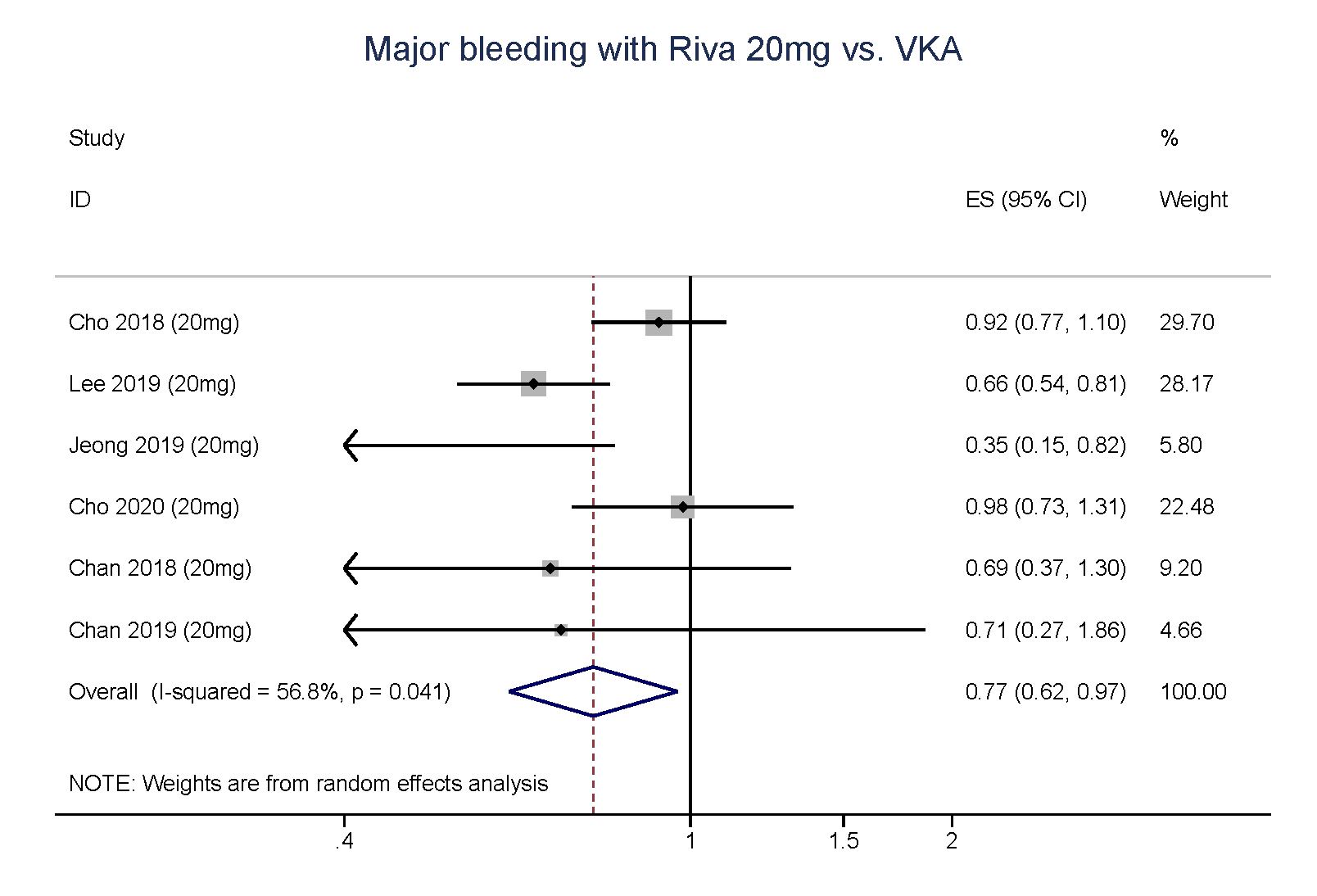


# Figure S11. Major bleeding with Riva 20mg vs. VKA (ES indicates hazard ratio)


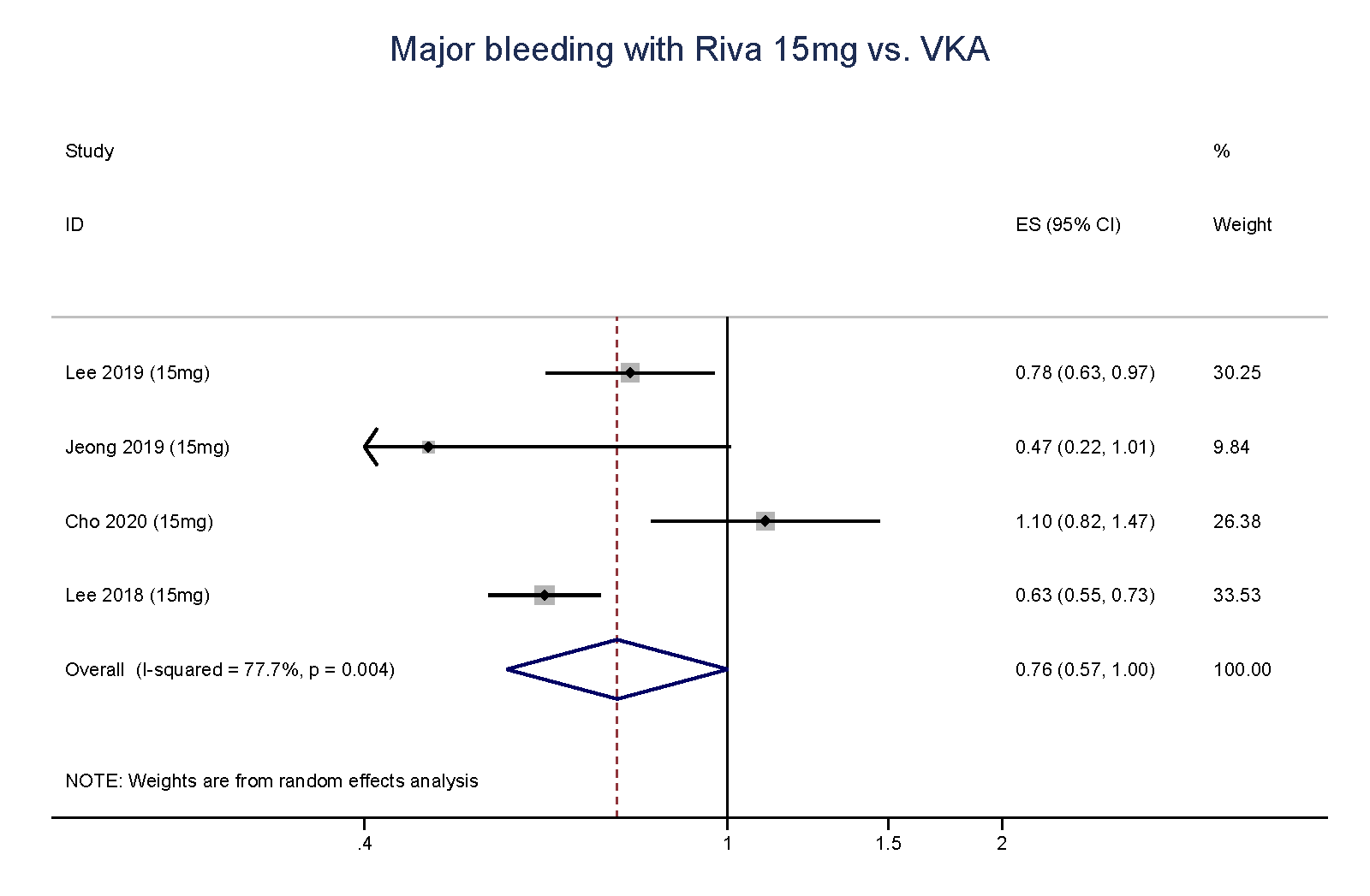


# Figure S12. Major bleeding with Riva 15mg vs. VKA (ES indicates hazard ratio)


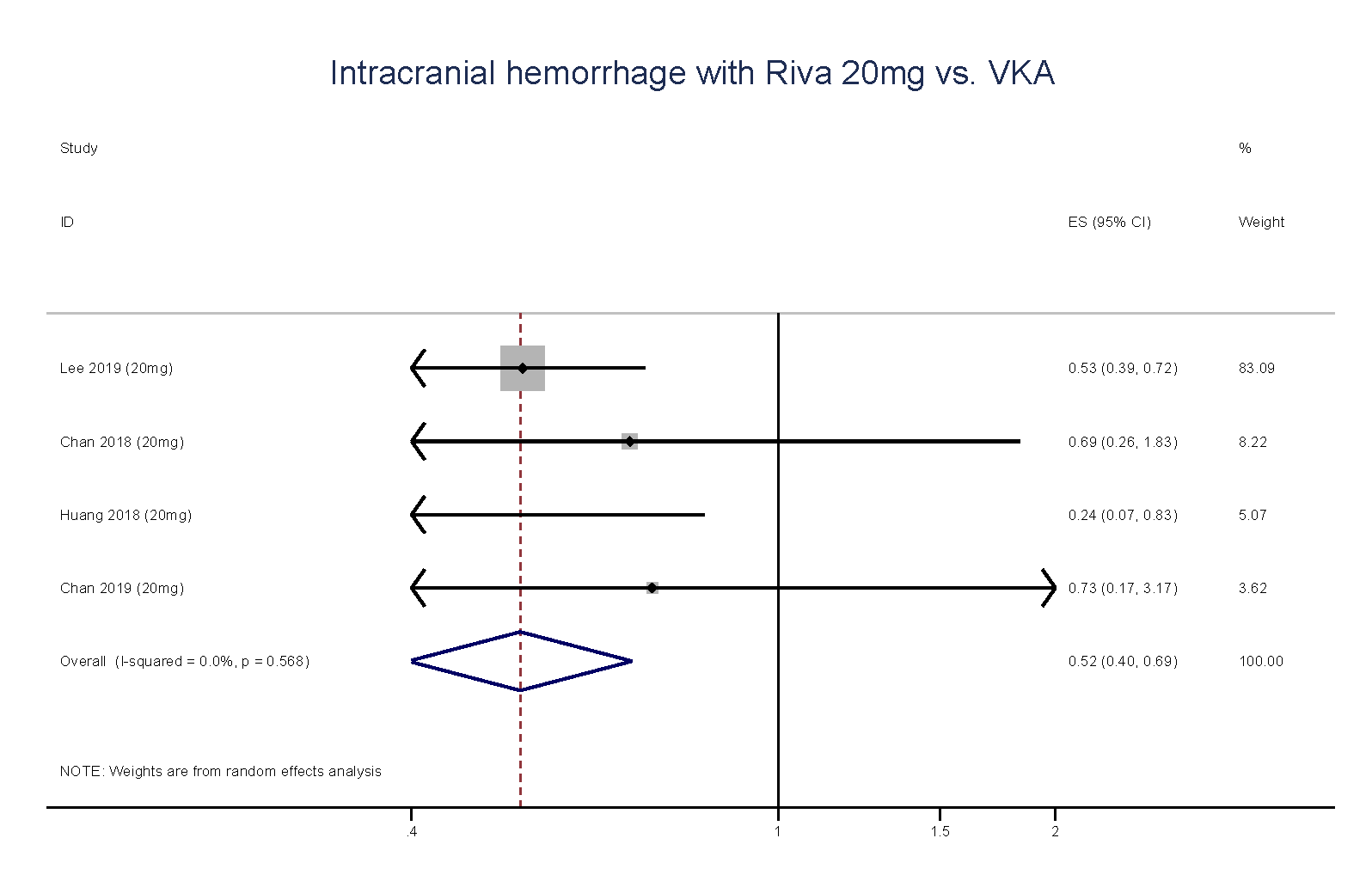


# Figure S13. Intracranial hemorrhage with Riva 20mg vs. VKA (ES indicates hazard ratio)


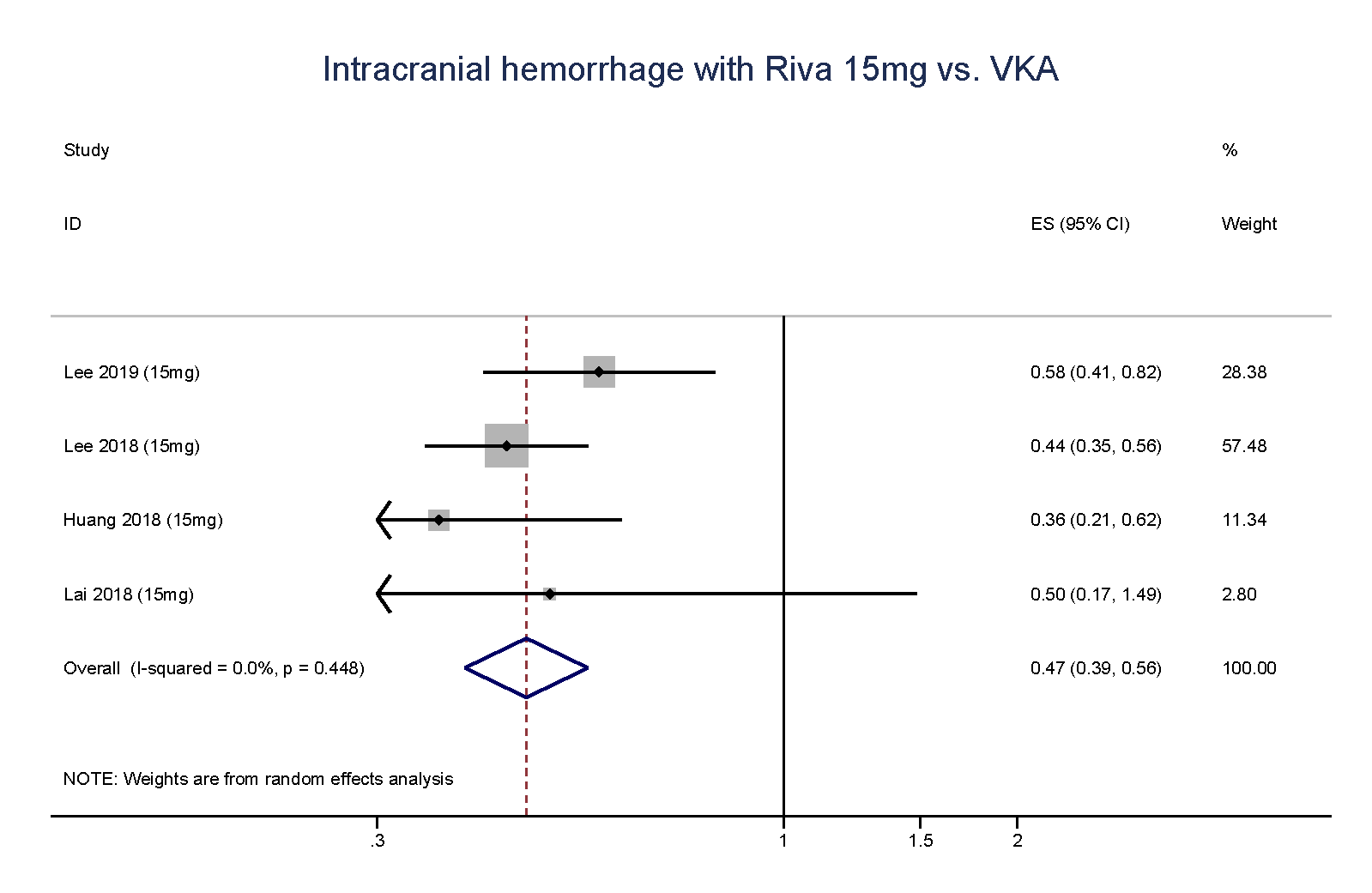


# Figure S14. Intracranial hemorrhage with Riva 15mg vs. VKA (ES indicates hazard ratio)


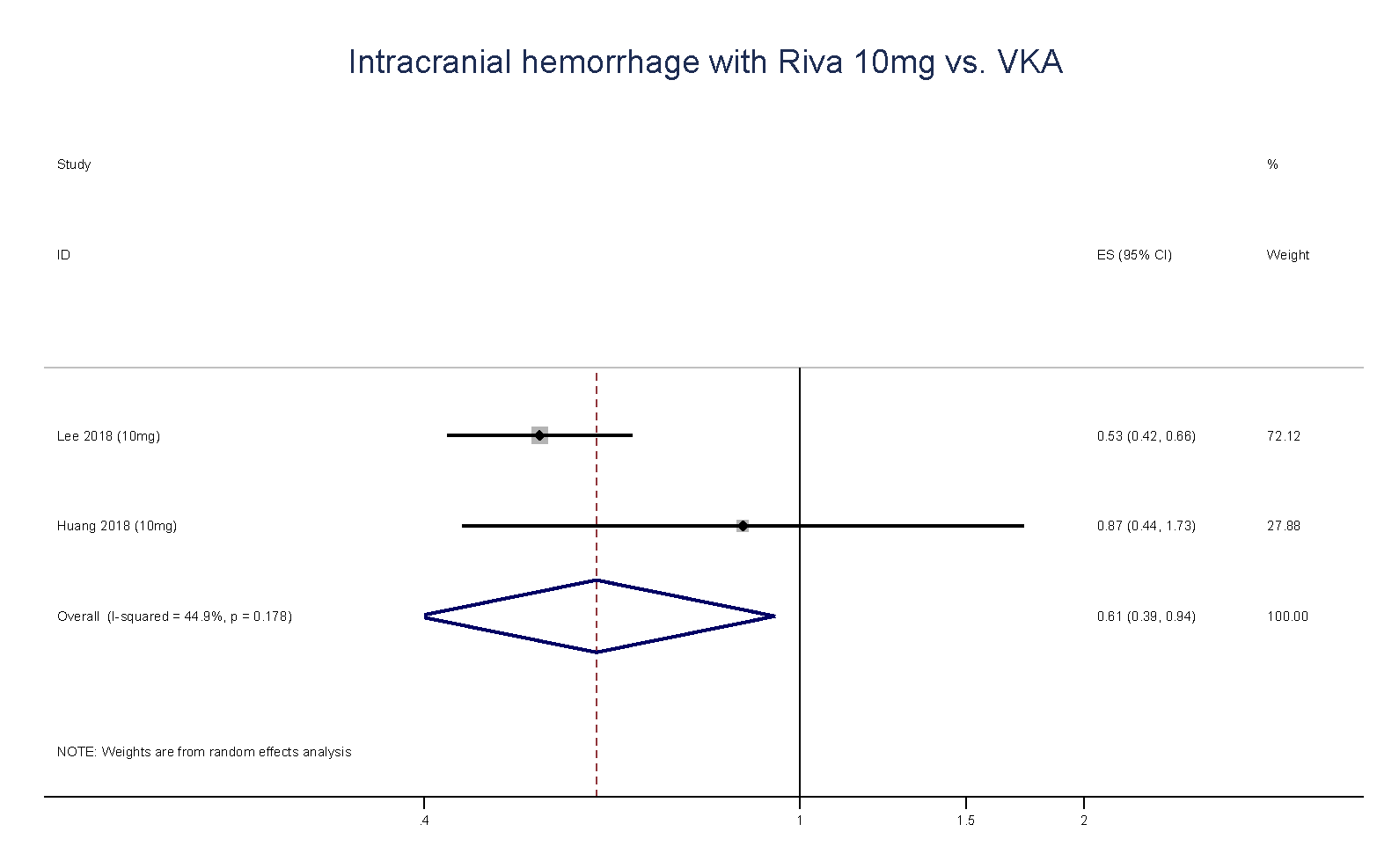


# Figure S15. Intracranial hemorrhage with Riva 10mg vs. VKA (ES indicates hazard ratio)


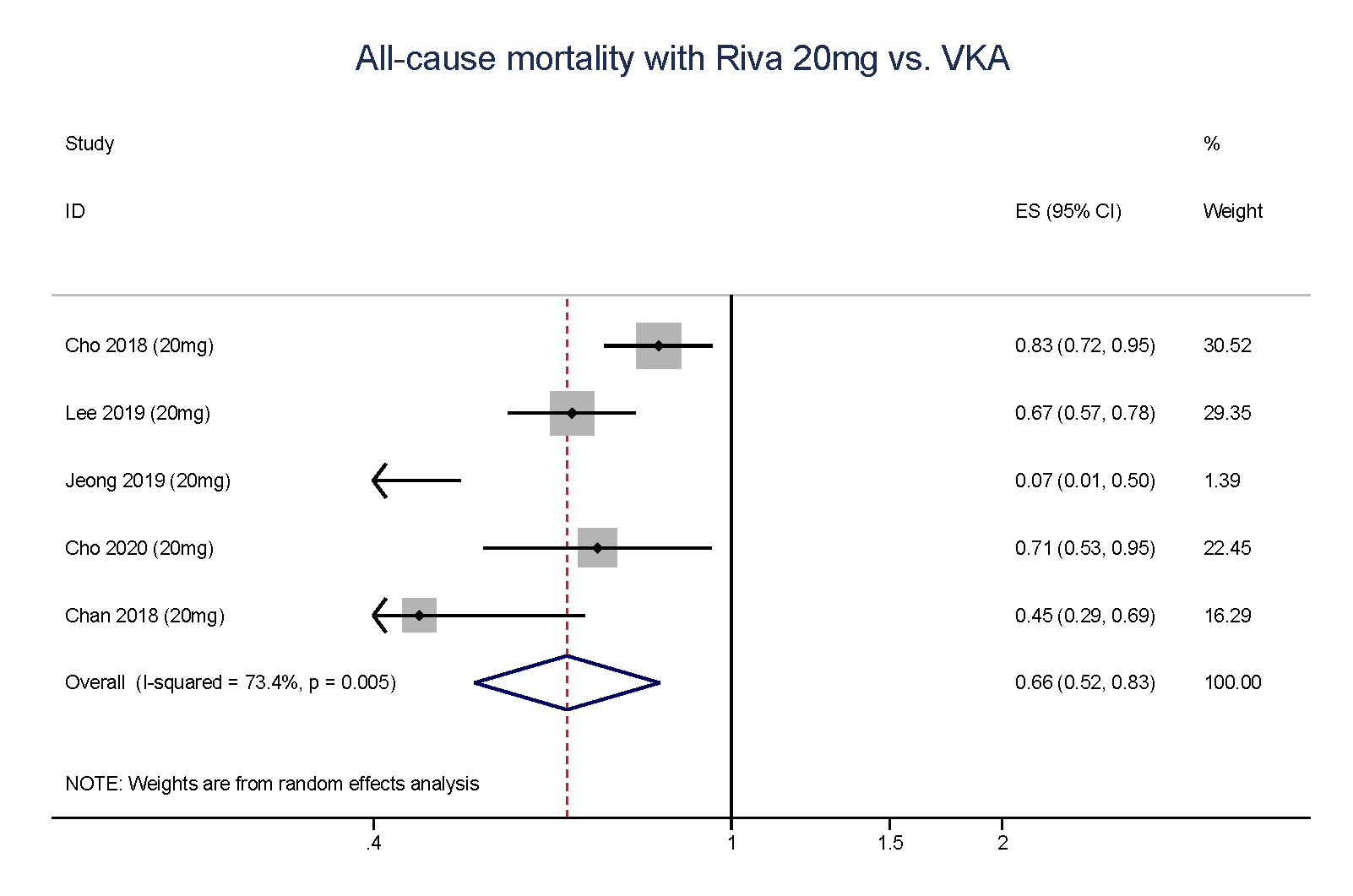


# Figure S16. All-cause mortality with Riva 20mg vs. VKA (ES indicates hazard ratio)


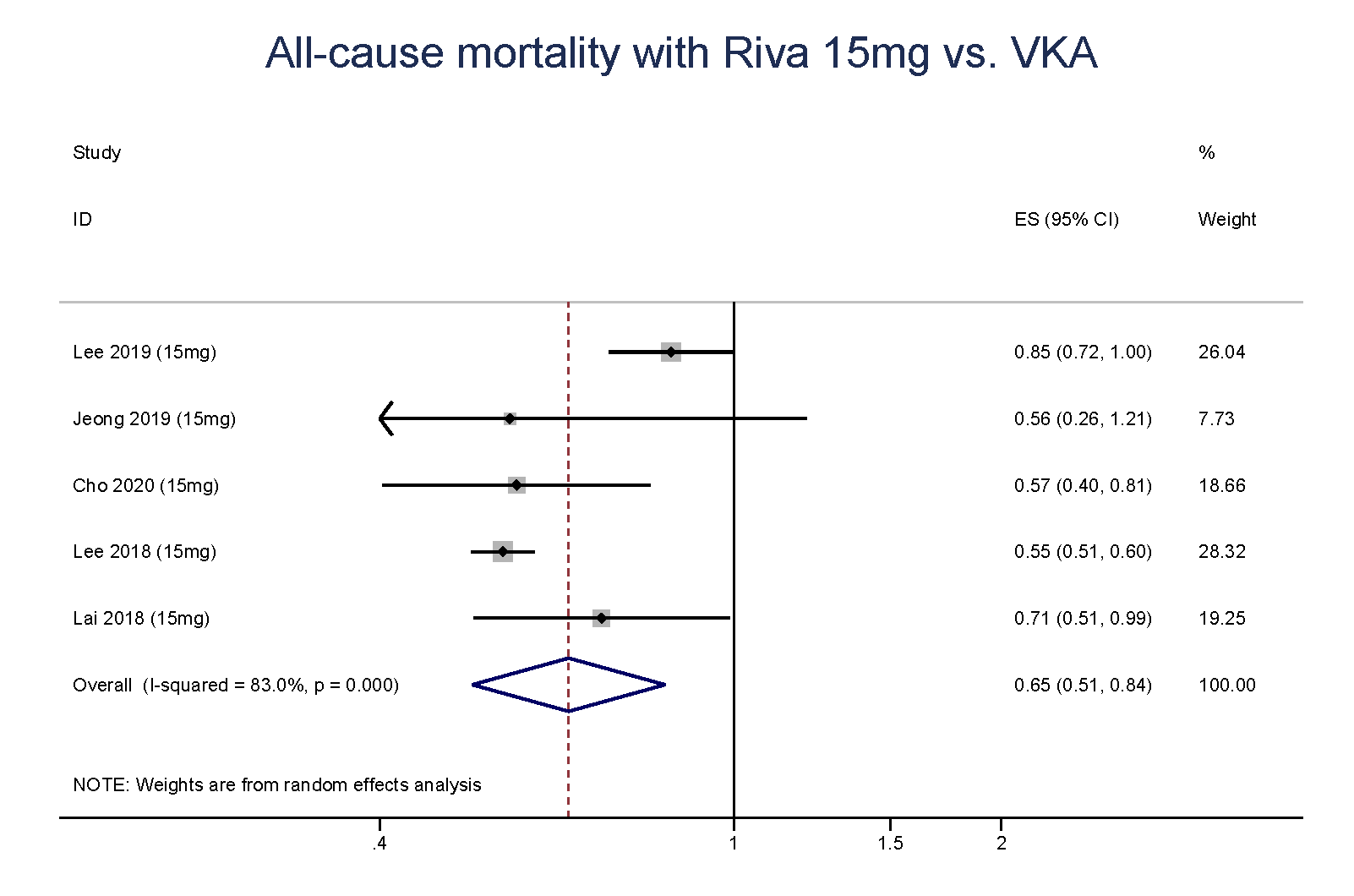


# Figure S17. All-cause mortality with Riva 15mg vs. VKA (ES indicates hazard ratio)


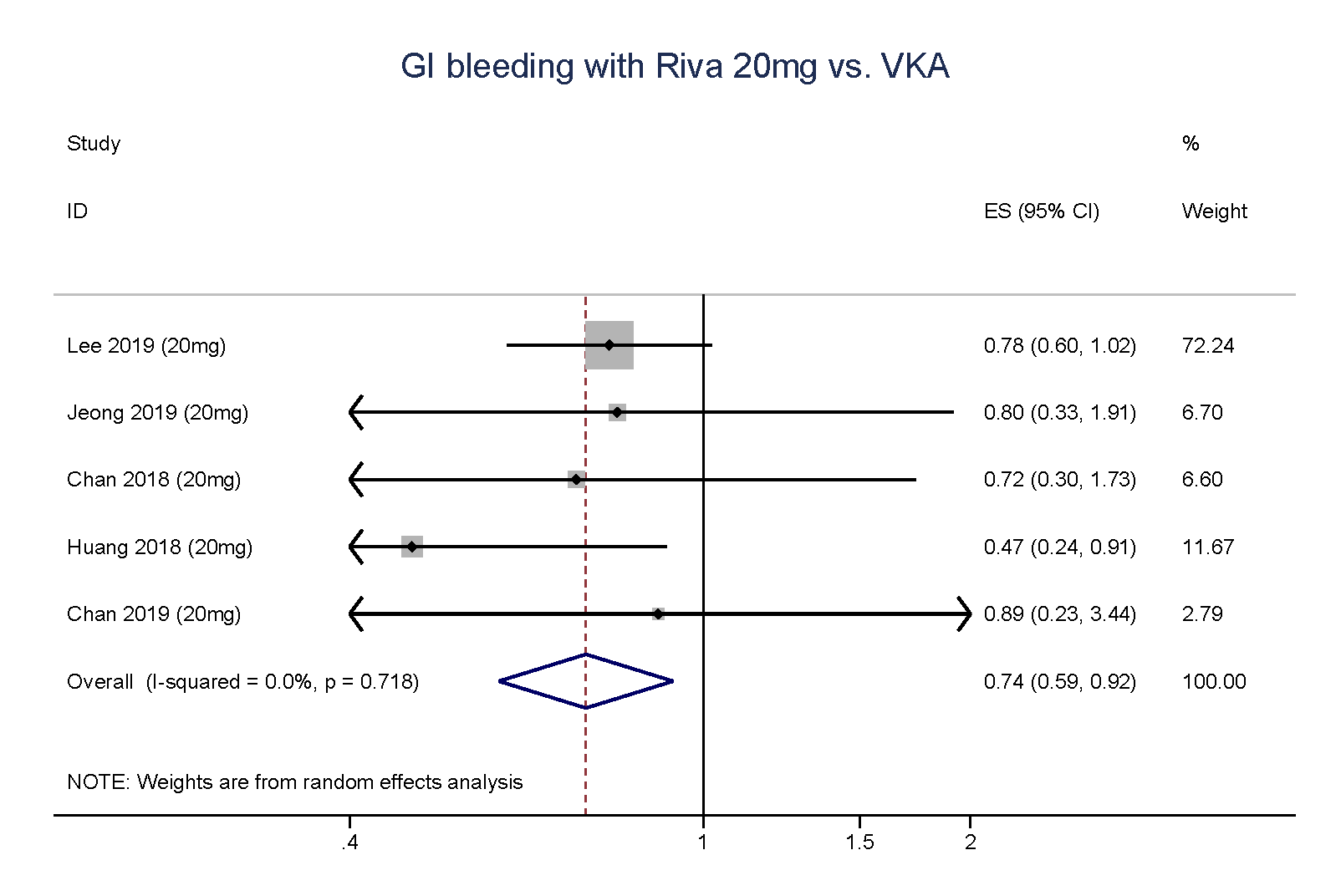


# Figure S18. GI bleeding with Riva 20mg vs. VKA (ES indicates hazard ratio)


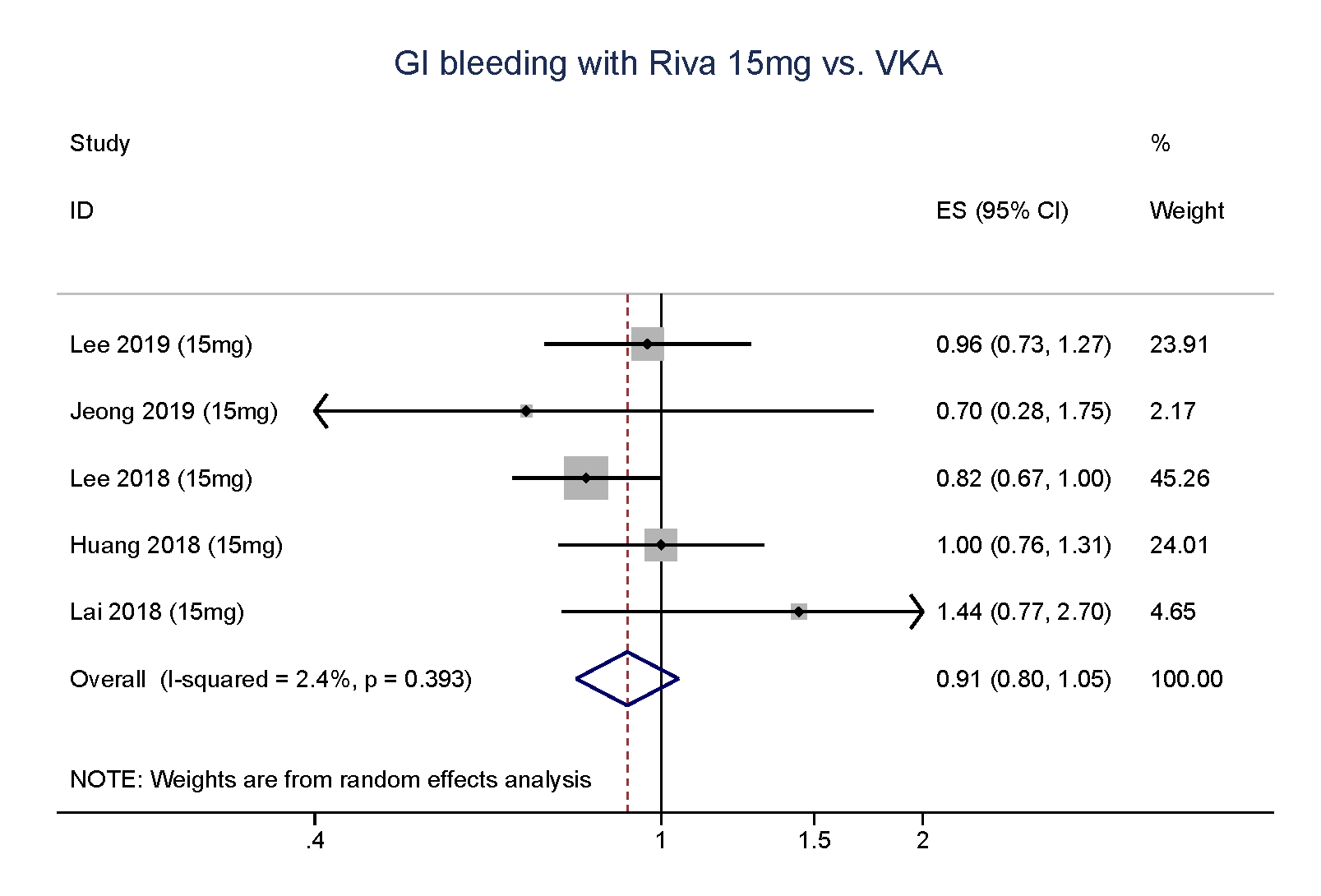


# Figure S19. GI bleeding with Riva 15mg vs. VKA (ES indicates hazard ratio)


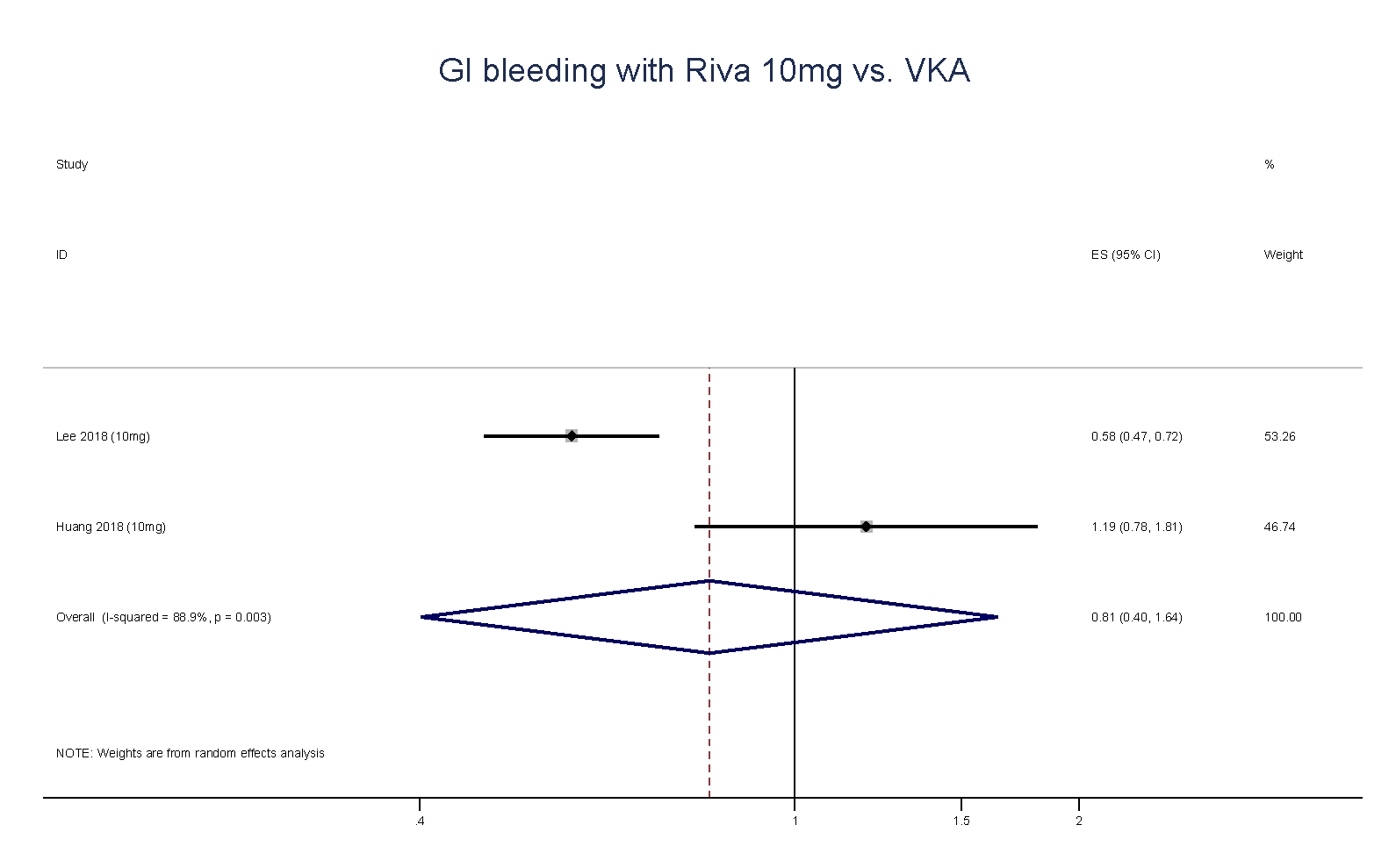


# Figure S20. GI bleeding with Riva 10mg vs. VKA (ES indicates hazard ratio)


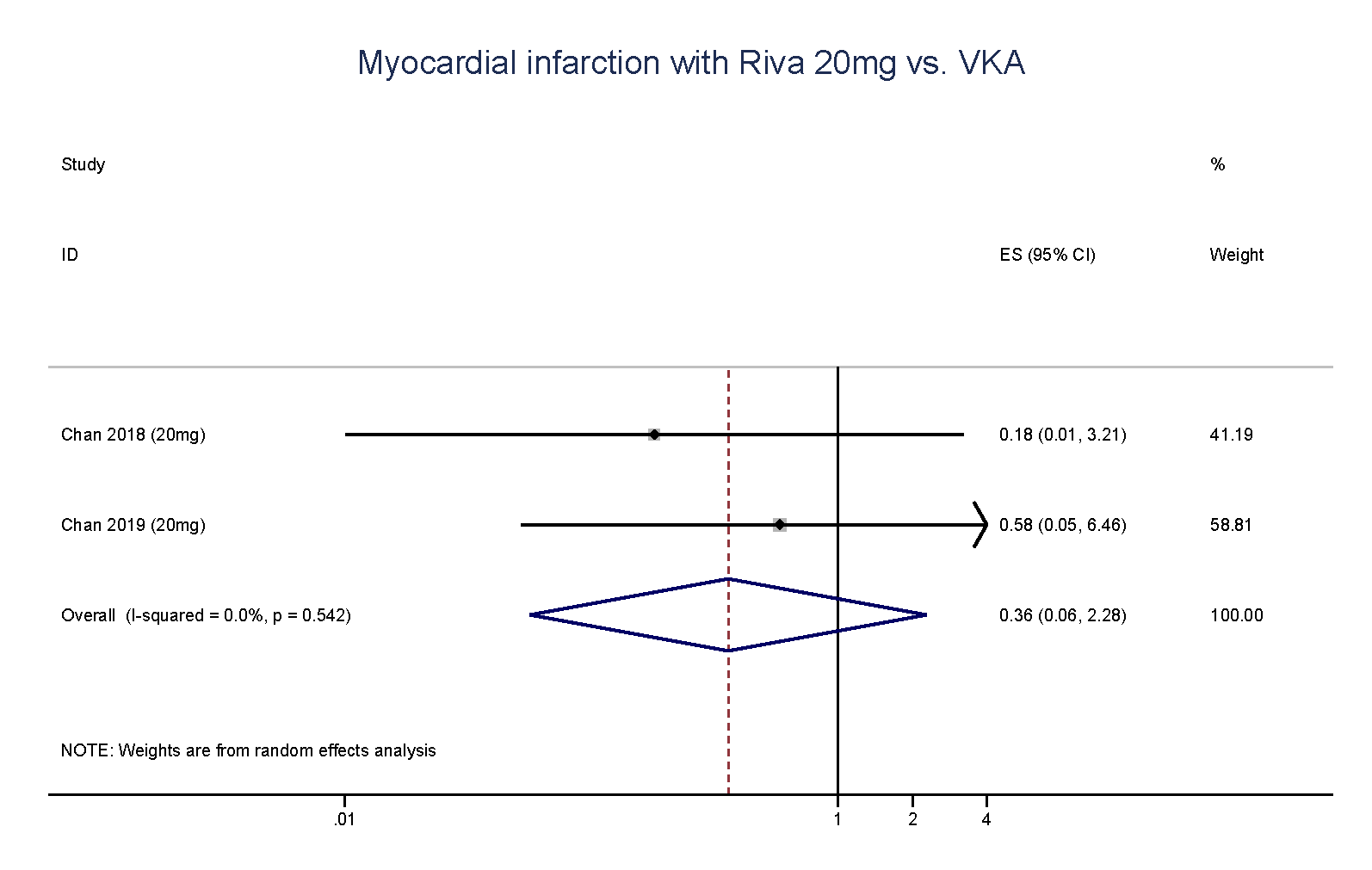


# Figure S21. Myocardial infarction with Riva 20mg vs. VKA (ES indicates hazard ratio)


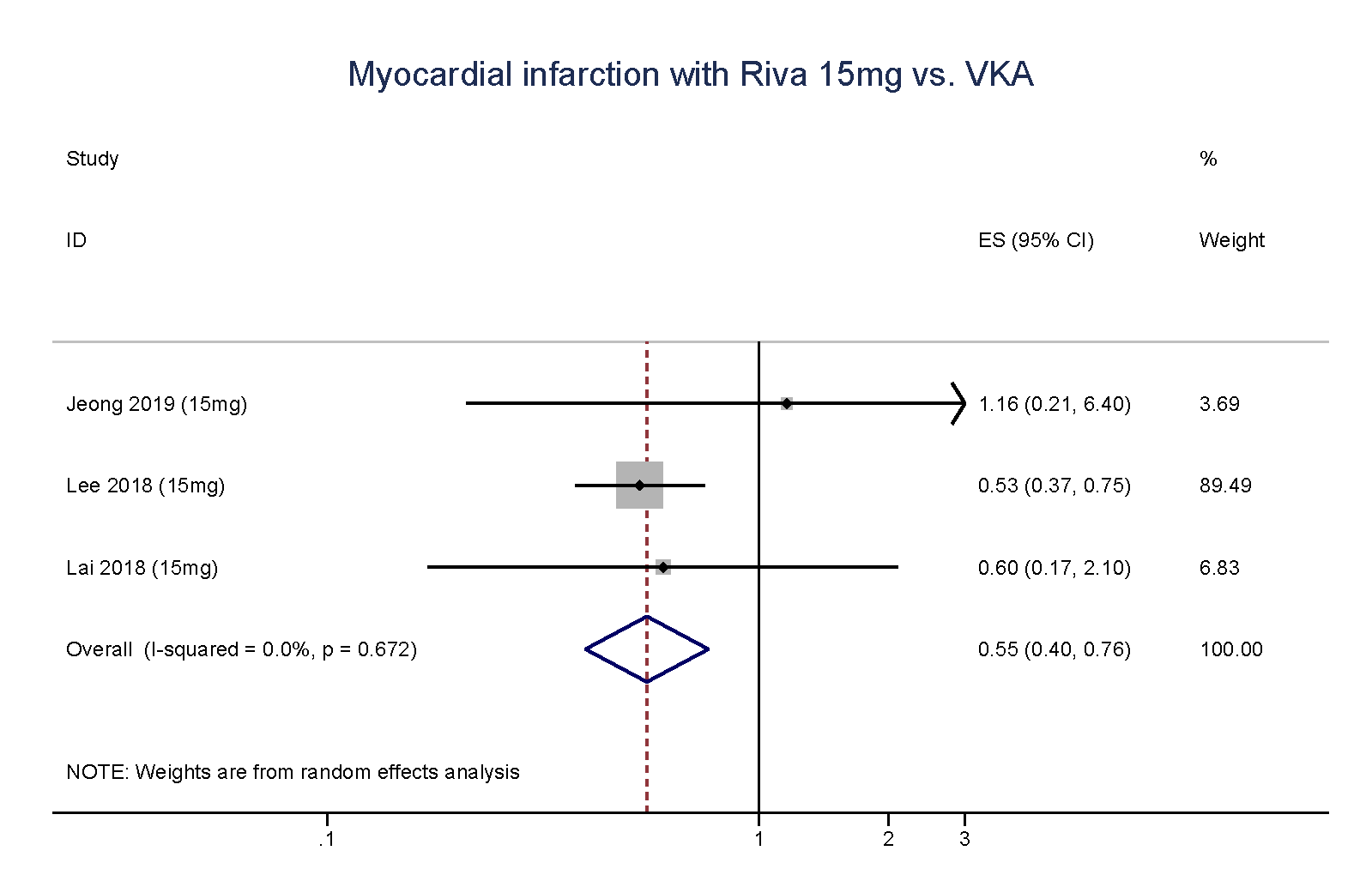


# Figure S22. Myocardial infarction with Riva 15mg vs. VKA (ES indicates hazard ratio)


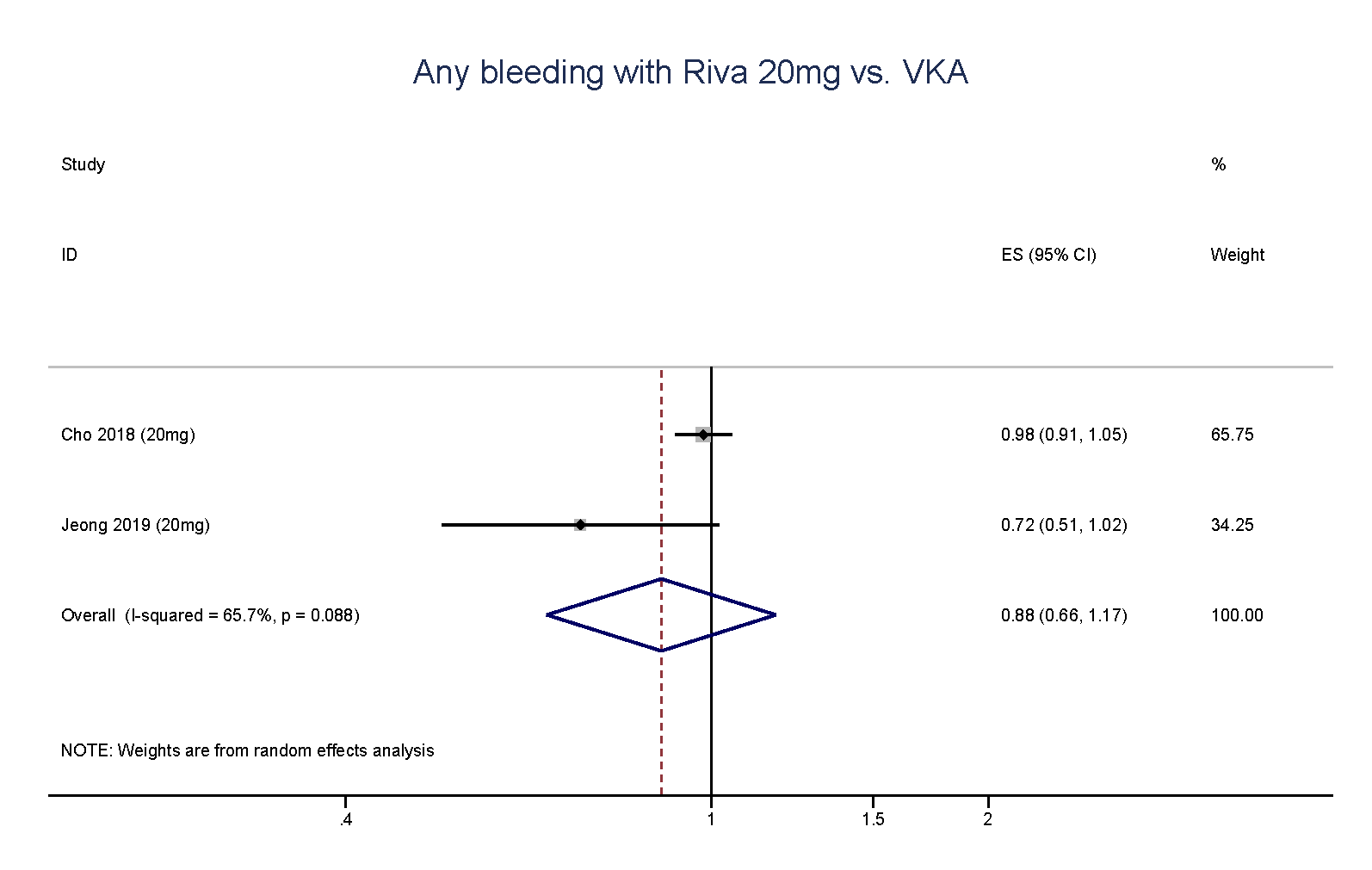


# Figure S23. Any bleeding with Riva 20mg vs. VKA (ES indicates hazard ratio)


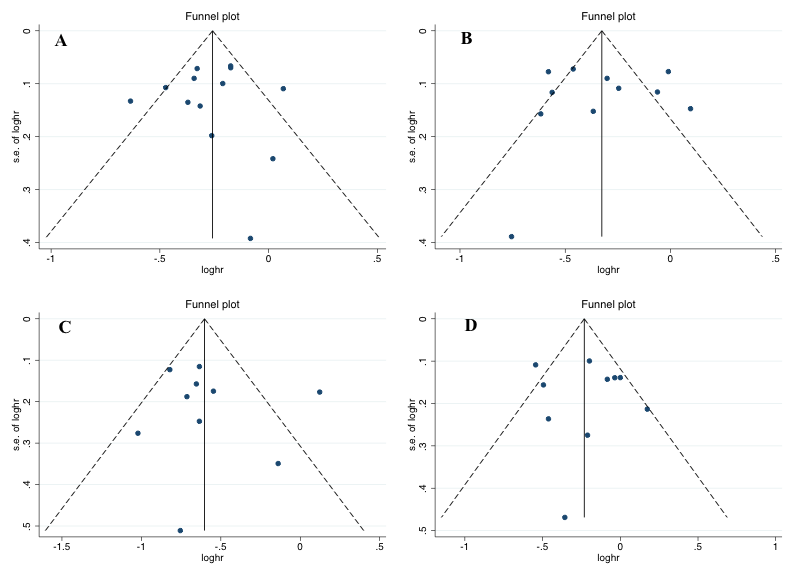


# Figure S24. Publication bias for reduced-dose of Rivaroxaban (A. Stroke/SE; B. MB; C. ICH; D. GIB)

**References:**

Arihiro, S., Todo, K., Koga, M., Furui, E., Kinoshita, N., Kimura, K., et al. (2016). Three-month risk-benefit profile of anticoagulation after stroke with atrial fibrillation: The SAMURAI-Nonvalvular Atrial Fibrillation (NVAF) study. *Int J Stroke* 11(5)**,** 565-574. doi: 10.1177/1747493016632239.

Bando, S., Nishikado, A., Hiura, N., Ikeda, S., Kakutani, A., Yamamoto, K., et al. (2018). Efficacy and safety of rivaroxaban in extreme elderly patients with atrial fibrillation: Analysis of the Shikoku Rivaroxaban Registry Trial (SRRT). *J Cardiol* 71(2)**,** 197-201. doi: 10.1016/j.jjcc.2017.08.005.

Cha, M.J., Choi, E.K., Han, K.D., Lee, S.R., Lim, W.H., Oh, S., et al. (2017). Effectiveness and Safety of Non-Vitamin K Antagonist Oral Anticoagulants in Asian Patients With Atrial Fibrillation. *Stroke* 48(11)**,** 3040-3048. doi: 10.1161/circoutcomes.119.005969

10.1161/strokeaha.117.018773.

Chan, Y.H., Kuo, C.T., Yeh, Y.H., Chang, S.H., Wu, L.S., Lee, H.F., et al. (2016). Thromboembolic, Bleeding, and Mortality Risks of Rivaroxaban and Dabigatran in Asians With Nonvalvular Atrial Fibrillation. *J Am Coll Cardiol* 68(13)**,** 1389-1401. doi: 10.1016/j.jacc.2016.06.062.

Chao, T.F., Chiang, C.E., Liao, J.N., Chen, T.J., Lip, G.Y.H., and Chen, S.A. (2020). Comparing the Effectiveness and Safety of Nonvitamin K Antagonist Oral Anticoagulants and Warfarin in Elderly Asian Patients With Atrial Fibrillation: A Nationwide Cohort Study. *Chest* 157(5)**,** 1266-1277. doi: 10.1016/j.chest.2019.11.025.

Cheng, W.H., Chao, T.F., Lin, Y.J., Chang, S.L., Lo, L.W., Hu, Y.F., et al. (2019). Low-Dose Rivaroxaban and Risks of Adverse Events in Patients With Atrial Fibrillation. *Drug Saf* 50(9)**,** 2574-2577. doi: 10.1007/s40264-019-00842-1

10.1161/strokeaha.119.025623.

Cirrone, F., Green, D., Papadopoulos, J., Chan, Y.H., Yeh, Y.H., Hsieh, M.Y., et al. (2018). The risk of acute kidney injury in Asians treated with apixaban, rivaroxaban, dabigatran, or warfarin for non-valvular atrial fibrillation: A nationwide cohort study in Taiwan. *J Thromb Thrombolysis* 265**,** 83-89. doi: 10.1007/s11239-017-1558-1

10.1016/j.ijcard.2018.02.075.

Ikeda, T., Ogawa, S., Kitazono, T., Nakagawara, J., Minematsu, K., Miyamoto, S., et al. (2019a). Outcomes associated with under-dosing of rivaroxaban for management of non-valvular atrial fibrillation in real-world Japanese clinical settings. *J Thromb Thrombolysis* 48(4)**,** 653-660. doi: 10.1007/s11239-019-01934-6.

Ikeda, T., Ogawa, S., Kitazono, T., Nakagawara, J., Minematsu, K., Miyamoto, S., et al. (2019b). Real-world outcomes of the Xarelto Post-Authorization Safety & Effectiveness Study in Japanese Patients with Atrial Fibrillation (XAPASS). *J Cardiol* 74(1)**,** 60-66. doi: 10.1016/j.jjcc.2019.01.001.

Lai, C.L., Chen, H.M., Liao, M.T., Lin, T.T., and Chan, K.A. (2017). Comparative Effectiveness and Safety of Dabigatran and Rivaroxaban in Atrial Fibrillation Patients. *J Am Heart Assoc* 6(4). doi: 10.1186/s12872-020-01340-4

10.1161/jaha.116.005362.

Lee, H.F., Chan, Y.H., Chang, S.H., Tu, H.T., Chen, S.W., Yeh, Y.H., et al. (2019a). Effectiveness and Safety of Non-Vitamin K Antagonist Oral Anticoagulant and Warfarin in Cirrhotic Patients With Nonvalvular Atrial Fibrillation. *Drugs R D* 8(5)**,** e011112. doi: 10.1007/s40268-019-0275-y

10.1161/jaha.118.011112.

Lee, H.F., See, L.C., Li, P.R., Liu, J.R., Chao, T.F., Chang, S.H., et al. (2021). Non-vitamin K antagonist oral anticoagulants and warfarin in atrial fibrillation patients with concomitant peripheral artery disease. *Eur Heart J Cardiovasc Pharmacother* 7(1)**,** 50-58. doi: 10.1093/ehjcvp/pvz072.

Lee, S.R., Choi, E.K., Han, K.D., Jung, J.H., and Oh, S. (2019b). Comparison of Once-Daily Administration of Edoxaban and Rivaroxaban in Asian Patients with Atrial Fibrillation. 9(1)**,** 6690. doi: 10.1038/s41598-019-43224-4.

Lee, S.R., Choi, E.K., Kwon, S., Han, K.D., Jung, J.H., Cha, M.J., et al. (2019c). Effectiveness and Safety of Contemporary Oral Anticoagulants Among Asians With Nonvalvular Atrial Fibrillation. *Stroke* 50(8)**,** 2245-2249. doi: 10.1161/strokeaha.119.025536.

Lee, S.R., and Lee, Y.S. (2019). Label Adherence for Non-Vitamin K Antagonist Oral Anticoagulants in a Prospective Cohort of Asian Patients with Atrial Fibrillation. 60(3)**,** 277-284. doi: 10.1016/j.ijcard.2018.03.060

10.3349/ymj.2019.60.3.277.

Li, H.J., Lin, S.Y., and Lin, F.J. (2021). Effectiveness and safety of non-vitamin K antagonist oral anticoagulants in Asian patients with atrial fibrillation and valvular heart disease. 1-14. doi: 10.1080/03007995.2021.1885365.

Li, W.H., Huang, D., Chiang, C.E., Lau, C.P., Tse, H.F., Chan, E.W., et al. (2017). Efficacy and safety of dabigatran, rivaroxaban, and warfarin for stroke prevention in Chinese patients with atrial fibrillation: the Hong Kong Atrial Fibrillation Project. *Clin Cardiol* 40(4)**,** 222-229. doi: 10.1161/circoutcomes.114.000907

10.1002/clc.22649.

Lin, Y.C., Chien, S.C., Hsieh, Y.C., Shih, C.M., Lin, F.Y., Tsao, N.W., et al. (2018). Effectiveness and Safety of Standard- and Low-Dose Rivaroxaban in Asians With Atrial Fibrillation. *J Am Coll Cardiol* 72(5)**,** 477-485. doi: 10.1016/j.jacc.2018.04.084.

Meng, S.W., Lin, T.T., Liao, M.T., Chen, H.M., and Lai, C.L. (2019). Direct Comparison of Low-Dose Dabigatran and Rivaroxaban for Effectiveness and Safety in Patients with Non-Valvular Atrial Fibrillation. *Acta Cardiol Sin* 35(1)**,** 42-54. doi: 10.18632/oncotarget.22026

10.6515/acs.201901_35(1).20180817a.

Mitsuntisuk, P., Nathisuwan, S., Junpanichjaroen, A., Wongcharoen, W., Phrommintikul, A., Wattanaruengchai, P., et al. (2020). Real-World Comparative Effectiveness and Safety of Non-Vitamin K Antagonist Oral Anticoagulants vs. Warfarin in a Developing Country. *Clin Pharmacol Ther*. doi: 10.2478/prilozi-2020-0032

10.1002/cpt.2090.

Miyamoto, S., Ikeda, T., Ogawa, S., Kitazono, T., Nakagawara, J., Minematsu, K., et al. (2020). Clinical Risk Factors of Thromboembolic and Major Bleeding Events for Patients with Atrial Fibrillation Treated with Rivaroxaban in Japan. *J Stroke Cerebrovasc Dis* 29(4)**,** 104584. doi: 10.1002/phar.1989

10.1016/j.jstrokecerebrovasdis.2019.104584.

Murakawa, Y., Ikeda, T., Ogawa, S., Kitazono, T., Nakagawara, J., Minematsu, K., et al. (2020). Impact of body mass index on real-world outcomes of rivaroxaban treatment in Japanese patients with non-valvular atrial fibrillation. *Heart Vessels* 35(8)**,** 1125-1134. doi: 10.1371/journal.pone.0195950

10.1007/s00380-020-01587-z.

Murata, N., Okumura, Y., Yokoyama, K., Matsumoto, N., Tachibana, E., Kuronuma, K., et al. (2019). Clinical Outcomes of Off-Label Dosing of Direct Oral Anticoagulant Therapy Among Japanese Patients With Atrial Fibrillation Identified From the SAKURA AF Registry. *Circ J* 83(4)**,** 727-735. doi: 10.1253/circj.CJ-18-0991.

Saito, K., Jenkins, A., Li, B., Mardekian, J., Terayama, Y., Kitazono, T., et al. (2020). Real-world outcomes of rivaroxaban treatment in elderly Japanese patients with nonvalvular atrial fibrillation. *Open Heart* 35(3)**,** 399-408. doi: 10.1136/openhrt-2019-001232

10.1007/s00380-019-01487-x.

Shim, J., On, Y.K., Kwon, S.U., Nam, G.B., Lee, M.H., Park, H.W., et al. (2020). A Prospective, Observational Study of Rivaroxaban For Stroke Prevention In Atrial Fibrillation - The XANAP Korea. *Korean J Intern Med*. doi: 10.1080/14740338.2019.1578344

10.3904/kjim.2020.217.

Shimokawa, H., Yamashita, T., Uchiyama, S., Kitazono, T., Shimizu, W., Ikeda, T., et al. (2018). The EXPAND study: Efficacy and safety of rivaroxaban in Japanese patients with non-valvular atrial fibrillation. *Int J Cardiol* 258**,** 126-132. doi: 10.1016/j.jstrokecerebrovasdis.2019.104584

10.1016/j.ijcard.2018.01.141.

Sugrue, A., Sanborn, D., Amin, M., Farwati, M., Sridhar, H., Ahmed, A., et al. (2020). Inappropriate Dosing of Direct Oral Anticoagulants in Patients with Atrial Fibrillation. *Am J Cardiol*. doi: 10.1093/ajhp/zxaa329

10.1016/j.amjcard.2020.12.062.

Tepper, P.G., Mardekian, J., Masseria, C., Phatak, H., Kamble, S., Abdulsattar, Y., et al. (2018). Real-world comparison of bleeding risks among non-valvular atrial fibrillation patients prescribed apixaban, dabigatran, or rivaroxaban. *PLoS One* 13(11)**,** e0205989. doi: 10.1371/journal.pone.0205989.

Tittl, L., Endig, S., Marten, S., Reitter, A., Beyer-Westendorf, I., and Beyer-Westendorf, J. (2018). Impact of BMI on clinical outcomes of NOAC therapy in daily care - Results of the prospective Dresden NOAC Registry (NCT01588119). *Int J Cardiol* 262**,** 85-91. doi: 10.1007/s00228-018-2540-3

10.1016/j.ijcard.2018.03.060.

Tsai, C.T., Liao, J.N., Chen, S.J., Jiang, Y.R., Chen, T.J., and Chao, T.F. (2021). Non-vitamin K antagonist oral anticoagulants versus warfarin in AF patients ≥ 85 years. e13488. doi: 10.1111/eci.13488.

Uchiyama, S., Atarashi, H., Inoue, H., Kitazono, T., Yamashita, T., Shimizu, W., et al. (2019). Primary and secondary prevention of stroke and systemic embolism with rivaroxaban in patients with non-valvular atrial fibrillation : Sub-analysis of the EXPAND Study. 34(1)**,** 141-150. doi: 10.1007/s00380-018-1219-0.

Umei, M., Kishi, M., Sato, T., Shindo, A., Toyoda, M., Yokoyama, M., et al. (2017). Indications for suboptimal low-dose direct oral anticoagulants for non-valvular atrial fibrillation patients. *J Arrhythm* 33(5)**,** 475-482. doi: 10.1016/j.joa.2017.05.008.

Yamashita, Y., Uozumi, R., Hamatani, Y., Esato, M., Chun, Y.H., Tsuji, H., et al. (2017). Current Status and Outcomes of Direct Oral Anticoagulant Use in Real-World Atrial Fibrillation Patients　- Fushimi AF Registry. *Circ J* 81(9)**,** 1278-1285. doi: 10.1253/circj.CJ-16-1337.

Zhao, J., Blais, J.E., Chui, C.S.L., Suh, I.H., Chen, E.Y.H., Seto, W.K., et al. (2020). Association Between Nonvitamin K Antagonist Oral Anticoagulants or Warfarin and Liver Injury: A Cohort Study. *Am J Gastroenterol* 115(9)**,** 1513-1524. doi: 10.1159/000502883

10.14309/ajg.0000000000000678.
